# Bayesian metamodeling of early T-cell antigen receptor signaling accounts for its nanoscale activation patterns

**DOI:** 10.3389/fimmu.2024.1412221

**Published:** 2024-10-25

**Authors:** Yair Neve-Oz, Eilon Sherman, Barak Raveh

**Affiliations:** ^1^ Racah Institute of Physics, The Hebrew University, Jerusalem, Israel; ^2^ School of Computer Science and Engineering, The Hebrew University of Jerusalem, Jerusalem, Israel

**Keywords:** T cell, immunological synapse, Bayesian metamodeling, T-cell activation, kinetic segregation, Lck, T-cell receptor, CD45

## Abstract

T cells respond swiftly, specifically, sensitively, and robustly to cognate antigens presented on the surface of antigen presenting cells. Existing microscopic models capture various aspects of early T-cell antigen receptor (TCR) signaling at the molecular level. However, none of these models account for the totality of the data, impeding our understanding of early T-cell activation. Here, we study early TCR signaling using Bayesian metamodeling, an approach for systematically integrating multiple partial models into a metamodel of a complex system. We inform the partial models using multiple published super-resolution microscopy datasets. Collectively, these datasets describe the spatiotemporal organization, activity, interactions, and dynamics of TCR, CD45 and Lck signaling molecules in the early-forming immune synapse, and the concurrent membrane alterations. The resulting metamodel accounts for a distinct nanoscale dynamic pattern that could not be accounted for by any of the partial models on their own: a ring of phosphorylated TCR molecules, enriched at the periphery of early T cell contacts and confined by a proximal ring of CD45 molecules. The metamodel suggests this pattern results from limited activity range for the Lck molecules, acting as signaling messengers between kinetically-segregated TCR and CD45 molecules. We assessed the potential effect of Lck activity range on TCR phosphorylation and robust T cell activation for various pMHC:TCR association strengths, in the specific setting of an initial contact. We also inspected the impact of localized Lck inhibition via Csk recruitment to pTCRs, and that of splicing isoforms of CD45 on kinetic segregation. Due to the inherent scalability and adaptability of integrating independent partial models *via* Bayesian metamodeling, this approach can elucidate additional aspects of cell signaling and decision making.

## Introduction

T cells orchestrate an immune response through the specific recognition of foreign antigens, which are presented on the surface of antigen-presenting cells (APCs). This interaction occurs at a highly specialized junction, termed the immune synapse (IS), between the T cell and the APC. The IS is characterized by its unique molecular architecture, where signaling molecules are dynamically recruited and organized (1). This organization is critical for the transduction of activation signals and subsequent regulation of the T cell response (2, 3). The formation of the IS is a pivotal event in the adaptive immune response, facilitating a targeted and effective reaction to foreign antigens.

**Figure 1:**
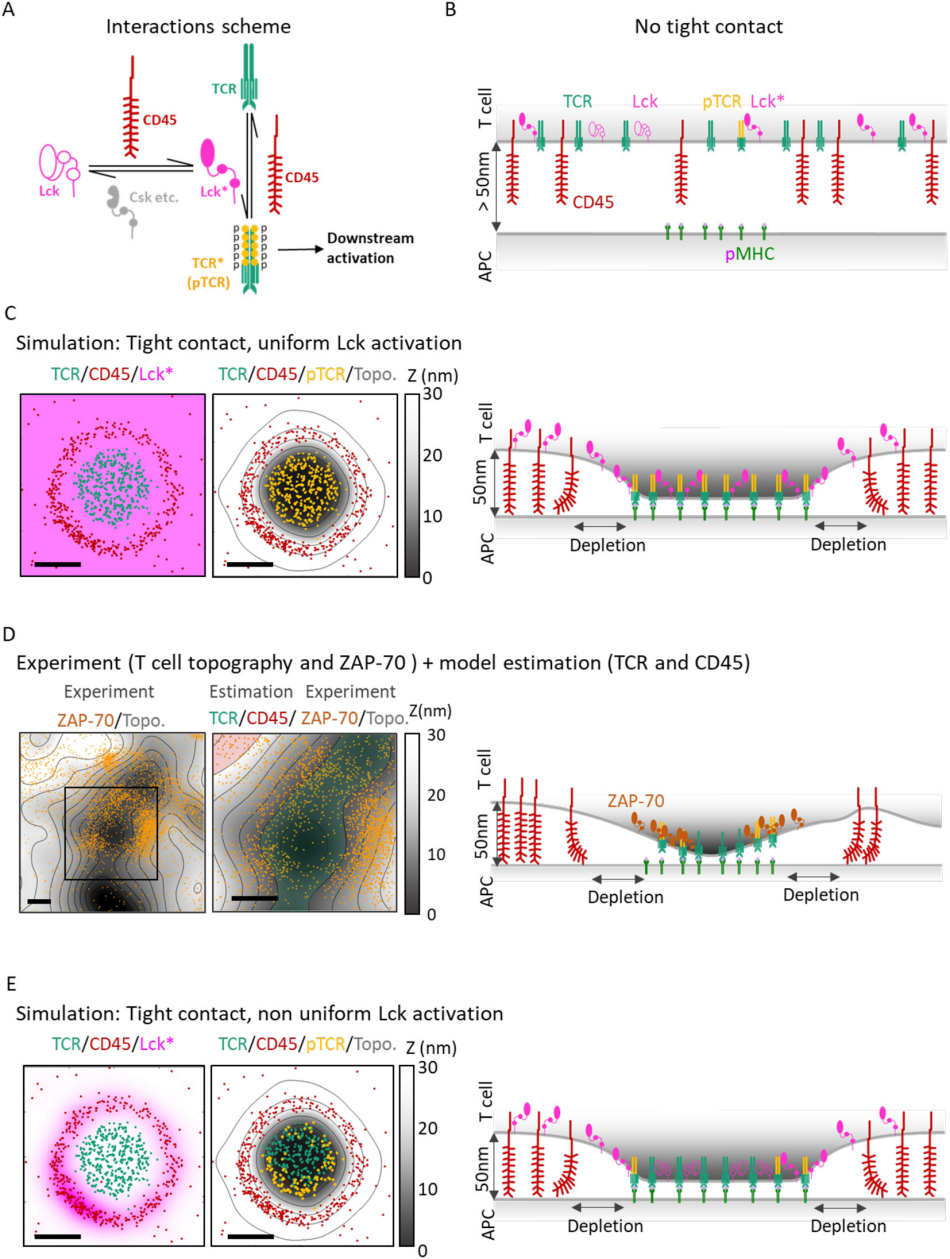
Overview: Integration of multiple molecular mechanisms is needed to qualitatively explain nanoscale TCR activation patterns at early contacts. **(A)** The molecular interactions considered in our model. CD45 deactivates the TCR (with its CD3 complex; TCR* or pTCR), turning it to a non-active form (TCR). CD45 also activates Lck to Lck*. The deactivation of Lck* to Lck is modeled implicitly, where various phosphatases and kinases (e.g. Csk; not an explicit part of the model) are known to deactivate Lck* (see Main Text). Lck* phosphorylates the TCR to pTCR (i.e. TCR*). Thus, CD45 acts both as a direct inhibitor and as an indirect enhancer of TCR phosphorylation. **(B)** Cartoon of a simplified side-view of a T-cell placed above an APC with no contact between them (z>50nm). TCR (green), CD45 (red), Lck* (full magenta), Lck (empty magenta) and phosphorylated TCR (orange). The molecules are represented as mixed, although in reality, they can be organized in separate or slightly mixed clusters. On the activating surface of the APC, the pMHC molecules (light green) are clustered. **(C)** left: A simulated 2µm x 2µm surface patch of a T cell forming a tight contact with an APC. Points show the locations of TCR (green) and CD45 (red). There is a blank ‘depletion zone’ between the TCR and the CD45 molecules. The magenta uniform color represents the Lck* distribution. center: The same molecular distribution in C-left with the added T cell membrane topography (gray colormap). The orange points show the locations of pTCR (TCR*) molecules that result from the Lck* distribution. TCR (including pTCR) are located mainly at z < 10nm. CD45 molecules are located mainly at 30nm < z < 40nm. right: Cartoon side-view of a T cell and APC in tight contact. TCR/pTCR molecules bind to the pMHC molecules, CD45 molecules are pushed to the sides and form a depletion zone. With Lck* uniformly scattered, the pTCR (TCR*) are uniformly phosphorylated. **(D)** left: 4µm x 4µm sample of experimental measurement of a T cell membrane topography (gray colormap) and ZAP-70 molecules (orange points). The ZAP-70 molecules are distributed around the lowest areas of the membrane where tight contact is formed. center: Zoom-in and added colored regions for estimated TCR (transparent green) and CD45 (transparent red) that marks higher probabilities of finding these molecules. right: Cross section of D-center with illustration of the interacting molecules. Green transparent area and red transparent area show the regions where we expect to find TCR and CD45 respectively at tight contact. Molecular distributions and membrane topography are identical to **(C)**. Here the Lck* distribution is a result of the sum of individual Lck* distributions where every distribution has a radial symmetry around a CD45 with exponential decay length of 70nm (left). The result (center, right) is a non-uniform distribution of pTCR where the pTCRs are located closer to the CD45 (center). Scale bars in **(C–E)** are 0.5μm.

The molecular signaling subprocesses integral to early T cell activation begin when T cell antigen receptors (TCRs; including the CD3 complex) encounter and bind to peptide-MHC (pMHC) complexes presented on antigen-presenting cells (APCs) (4). This binding triggers a cascade of downstream signaling pathways, but only if the peptide is identified as a cognate foreign antigen. A critical early event in this cascade is the phosphorylation of immunoreceptor tyrosine-based activation motifs (ITAMs) located in the cytoplasmic domains of the TCRs (**Figure 1A**). This phosphorylation event is tightly regulated since it significantly amplifies and relays the signal, leading to diverse macroscopic effector functions (5, 6). The phosphatase CD45, a surface glycoprotein, plays a dual role in this context. On one hand, it dephosphorylates ITAMs, thus attenuating TCR signaling (7) and consequently, preventing T cell activation (8, 9). On the other hand, CD45 paradoxically also promotes T cell activation by dephosphorylating an inhibitory site on the kinase Lck, part of the c-Src family (10) (**Figure 1A**). Once this inhibitory site is dephosphorylated, Lck undergoes auto-phosphorylation, becoming active. The active Lck then phosphorylates the TCR ITAMs. The intricate balance between these opposing regulatory mechanisms by CD45 is a crucial early checkpoint in deciding whether a T cell will be activated or not (4).

Recent studies have identified multiple nanoscale patterns of signaling molecules at the IS, such as molecular clustering (11, 12). The spatiotemporal organization of these molecules is affected by their relative sizes, elasticity, their binding properties to other surface and cytoplasmic molecules, the presence and locations of these other molecules, the topography of the plasma membrane, and the stage of IS formation (13). Prior to T cell-APC binding, TCR and CD45 molecules are distributed across the T cell membrane (**Figure 1B**), although some pre-organization may occur at the tips of microvilli (14). Upon TCR recognition of cognate pMHCs on APCs, a tight contact forms between the two cells, eventually leading to a mature IS characterized by concentric rings of supramolecular activation clusters (SMACs) (1, 15). Early in this contact, pronounced TCR clusters form (16), inducing local separation of bulky glycoproteins like CD45 from the TCR clusters (17–19) (**Figure 1C**, right panel). The physical segregation of CD45 from TCR has been suggested to promote TCR signaling downstream, leading to T cell activation, by preventing the phosphatase activity of CD45 from quenching the signaling of TCRs through their phosphorylated ITAMs (pTCRs) (13). This mechanism is known as the kinetic segregation (KS) model. Thus, nanoscale spatiotemporal organization of molecules at the IS directly influences TCR signaling and response.

While microscopic mechanisms such as the KS model rationalize the formation of certain nanoscale patterns (13, 18–21), the mechanisms underlying other key observed nanoscale patterns and their implications on signaling remain undercharacterized. For instance, if one assumes that Lck molecules diffuse freely in active form after dephosphorylation by CD45 (22) (**Figure 1C**, left panel), an immediate consequence would be a uniform activation pattern for nearby TCRs (**Figure 1C**, middle and right panels). However, such a uniform activity pattern for Lck is inconsistent with empirical evidence: while it is challenging to directly monitor TCR activation, Razvag et al. (23) measured the distribution of the pTCR-binding tyrosine kinase ZAP-70 in relation to the membrane surface topography of live T cells, as they engage TCR-stimulating coverslips. ZAP-70 served as a marker for pTCRs in that experiment since it is quickly recruited to the phosphorylated ITAM motifs on $\zeta_{1}$ chains upon TCR activation (24, 25). For imaging ZAP-70 simultaneously with the membrane topography, photoactivated localization microscopy (PALM) was employed concurrently with internal reflectance microscopy (IRM). Surprisingly, such super-resolution imaging showed that ZAP-70 molecules, and by proxy, pTCRs, are enriched at the surrounding slopes of the tight contact of the plasma membrane (**Figure 1D**, left). In other words, the observed activity pattern of Lck, leading to TCR phosphorylation, is not uniform (as in **Figure 1C**).

To quantitatively characterize the mechanism leading to the observed nanoscale patterns of signaling at the early-forming IS, we created a model of early T-cell activation. We first investigate what modeling assumptions are necessary and sufficient to explain the observed patterns based on the relevant theoretical and experimental observations. Given the complexity of early T-cell activation, we employ our recent Bayesian metamodeling approach (26). In Bayesian metamodeling, the modeling of a complex system or process is broken into several independent modeling tasks, resulting in a set of partial models, each describing some aspect of the modeled system or process; the partial models are then combined and harmonized in a unified metamodel of the entire process using the principles of Bayesian statistics.

Here, we created a metamodel of T-cell activation that combines three partial models corresponding to key steps in the early engagement of T cells and APCs. The first partial model describes the kinetic segregation (KS model) between TCR and CD45 molecules (13), the second model describes the spatiotemporal distribution of Lck in its active form (Lck*) (Lck-A model) (27), and the third model describes the phosphorylation pattern of the TCR ITAMs (pTCR model) (5). We show that on their own, none of these models correctly accounts for the observed nanoscale organization of these signaling molecules at the nascent IS. However, the unified metamodel combines information to correctly predict these unique nanoscale patterns, and inform the molecular mechanisms through which those patterns emerge. We then studied the relevance and outcomes of our model under multiple physiological scenarios, including a range of pMHC: TCR association strengths as these molecules interact in an initial contact, Csk recruitment to pTCRs, and that of splicing isoforms of CD45.

## Results

**Figure 2:**
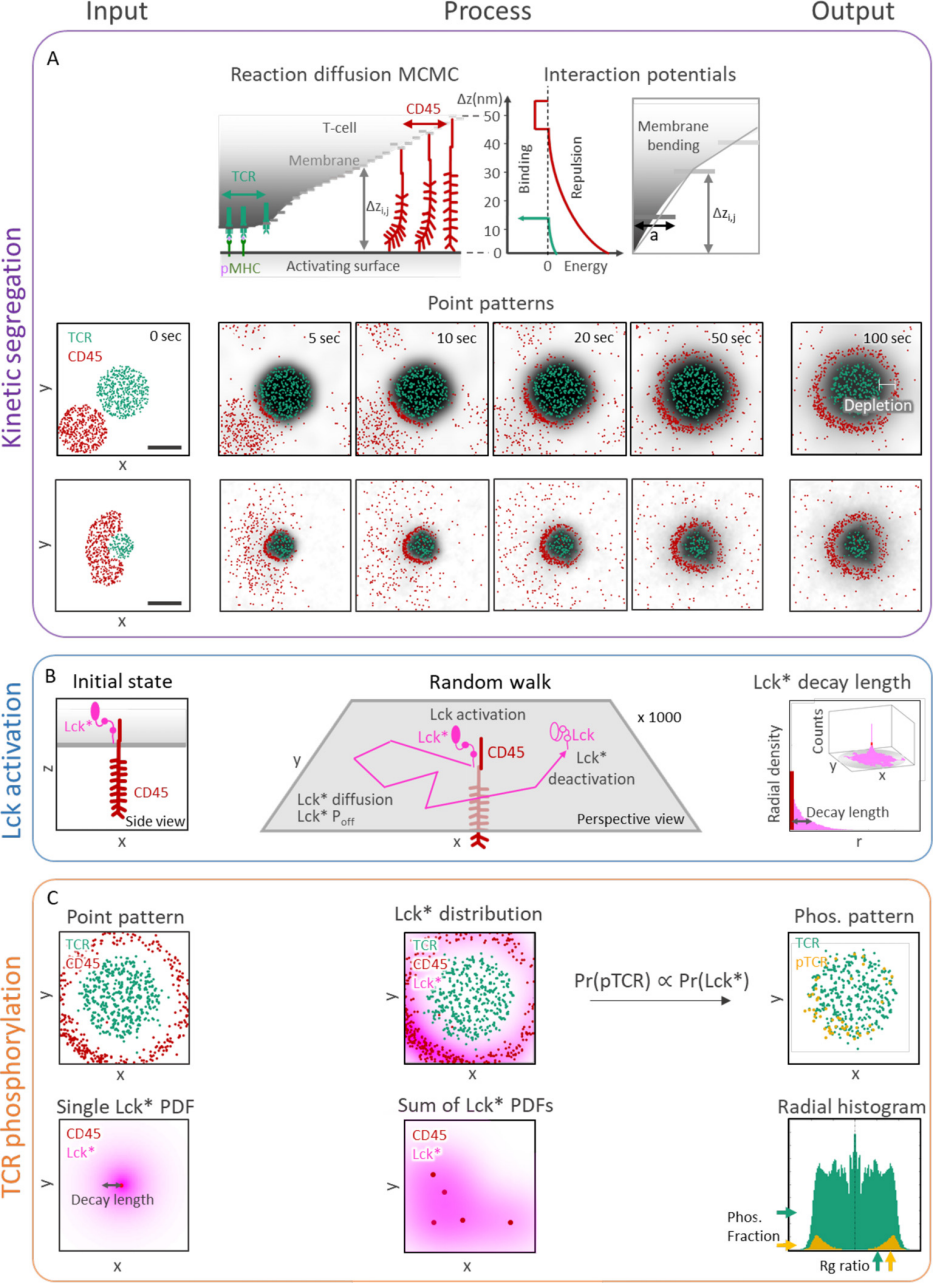
Stochastic modeling and simulations yield TCR activation patterning at early contacts. **(A)** Kinetic segregation (KS) model. Top row: Cartoon side view of interactions during the simulation. TCR binds to pMHC, CD45 pushes the activating surface and weakly binds to it (implicitly accounting for other cell-cell adhesion forces). Molecules diffuse under constraints of the Markov Chain Monte Carlo (MCMC) simulation. The interaction potentials are visualized on the right. Bottom rows: Samples of a simulation result at different times (0, 5, 10, 20, 50, and 100 sec). The distance between TCRs and CD45s (white arrow) marks the width of a depletion zone, where both molecules are absent. Scale bar in **(A)** is 0.5μm. **(B)** Lck activation (Lck-A) model. left: Side view scheme of a T cell membrane with a transmembrane CD45 and an Lck*. center: Perspective scheme of a random walk of Lck*. The Lck* begins its random walk path upon encountering a CD45 molecule and spontaneously being deactivated (to Lck) with probability *P_off_* per second. right: a perspective view (inset) of 1000 random walk paths of Lck* originated at CD45. The distribution height is the number of counts inside a 2D array of bins. The results are summarized as 1D radial histogram of Lck* count as a function of distance from the CD45 at the end of their random walk path. The result is a characteristic ‘decay-length’ of Lck*. **(C)** TCR phosphorylation (pTCR) model. left, top: point pattern of TCR and CD45 molecules. left, bottom: 2D radial probability density function of Lck* with an exponential decay around a single CD45. center, top: accumulated PDFs of Lck* around every CD45 of the point pattern from C left, top. center, bottom: sum of 2D radial distribution functions of Lck*. right, top: The resulting pTCR (yellow points) and TCR (green points). right, bottom: A radial histogram of the point pattern. The histogram is symmetric around its center. The horizontal arrows on the right mark the level of TCR (green) and pTCR (yellow). The vertical arrow at the bottom marks the TCRs’ and pTCRs’ radius of gyration.

**Figure 3:**
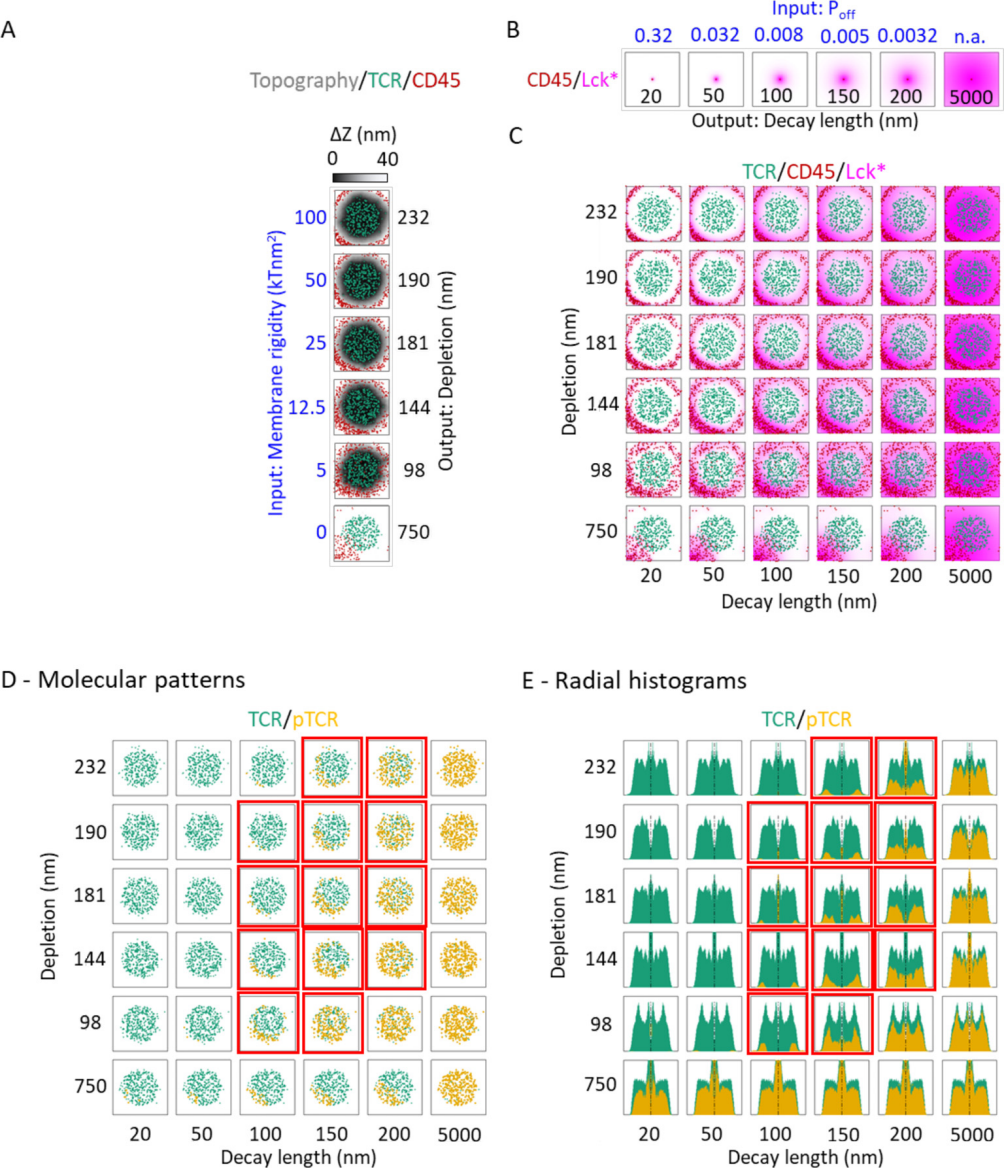
Plasma membrane rigidity and decay-length of active Lck modulate pTCR patterning. **(A)** KS model outputs showing the membrane topography (gray colormap) together with the spatial distribution of TCR (green) and CD45 (red) at t = 100 sec, simulated using different input values for the plasma membrane rigidity input parameter (left axis, blue labels). The black labels on the right axis indicate different output depletion widths, quantifying the segregation between TCR and CD45 molecules, computed from their spatial distributions. **(B)** Lck-A model outputs showing the distribution of Lck* (magenta) about a single CD45 molecule, computed using different input values for Lck* deactivation (top axis, blue labels). The black labels on the bottom axis indicate different output decay-length values. **(C)** Superposition of the outputs of the KS model as in **(A)** and the Lck-A model as in **(B)** for different combinations of their respective input parameters. The Lck* distribution is computed from the sum of all the individual distributions about every individual CD45 molecule. **(D)** The same array presented in **(C)**, but with pTCR distributions computed by the pTCR model using the corresponding TCR and Lck-A distributions from **(C)** as inputs. The red frames, here and in **(E)**, mark regions where the simulation results (qualitatively) resemble the experimental results. **(E)** Array of histograms of the molecular distributions in **(D)**.

**Figure 4:**
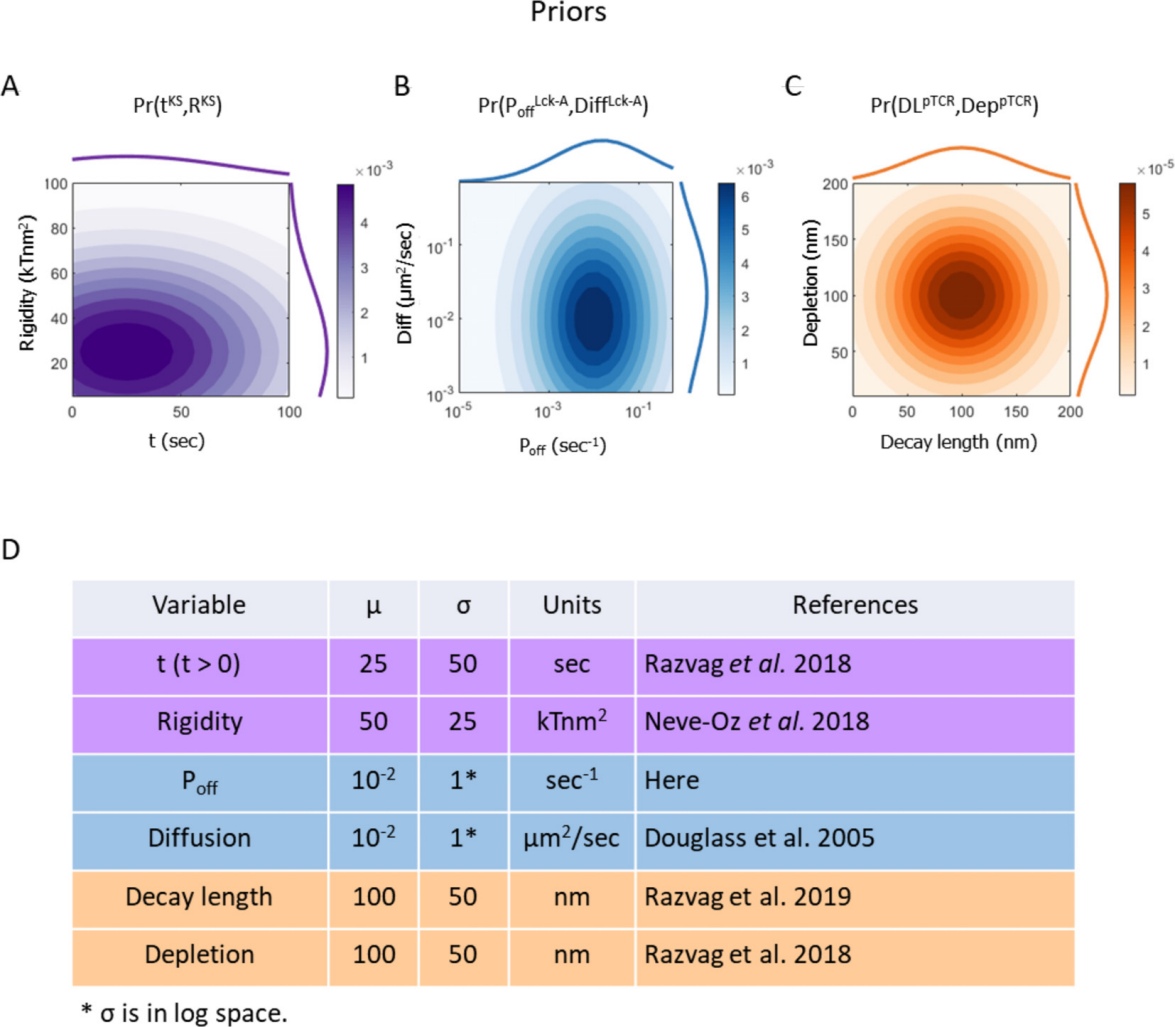
Prior probability density functions (PDF) of the models’ free parameters. Superscripts in variable names indicate the model to which they belong. **(A)** Prior PDF of time from activation onset t^KS^ (top histogram), rigidity parameter R^KS^ (right histogram) and their joint PDF (filled contours) in the KS model. **(B)** Prior PDF of Lck* deactivation rate P_off_^Lck-A^ (top histogram), diffusion coefficient Diff^Lck-A^ (right histogram) and their joint PDF (filled contours) in the Lck-A model. **(C)** Prior PDF of Lck* decay length DL^pTCR^ (top histogram), depletion width Dep^pTCR^ (right curve) and their joint PDF (filled contours) in the pTCR model. **(D)** Mean and standard deviation of the prior PDFs for key model variables in the surrogate KS model (magenta), Lck-A model (blue) and pTCR model (orange). These priors are intended as rough estimates, based on prior measurements, when possible, but with sufficiently wide margins to cover a wide range of reasonable value ranges; in subsequent metamodeling steps, these priors are updated to infer more accurate and precise posterior estimates based on observed data and propagation of information between the different models (26).

**Figure 5:**
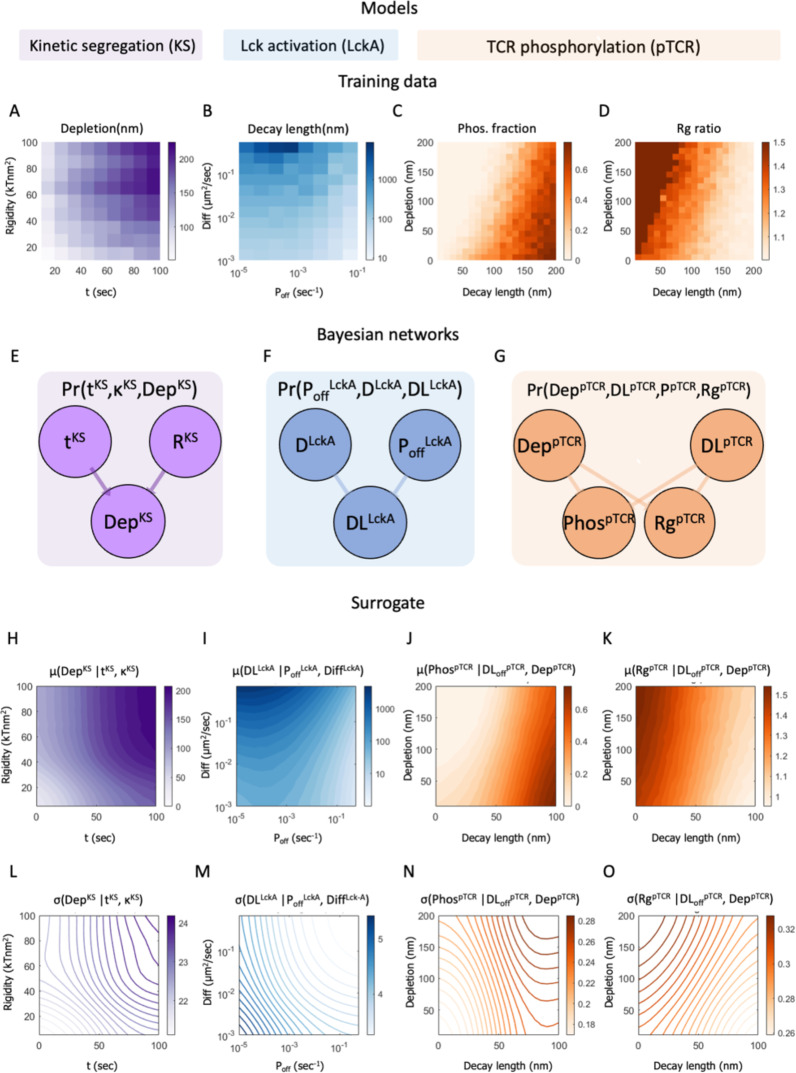
Construction of the surrogate models. **(A–D)** Training data for fitting a surrogate model. **(A)** Heatmap of the training data for the KS (kinetic segregation) model, showing the width of the depletion zone between TCR and CD45 molecules (Dep^KS^) computed using the KS model as a function of time (t^KS^) and membrane rigidity, (R^KS^). **(B)** Heatmap of the training data for the Lck-A (Lck activation) model, showing the decay length of Lck* (DL^Lck-A^) computed using the Lck-A model as a function of Lck deactivation rate (P_off_) and Lck diffusion coefficient Diff (μm^2^/sec). **(C, D)** Heatmap of the training data for the pTCR (TCR phosphorylation) model, showing the phosphorylation ratio Phos (in **C**) and the ratio between the RMSD of pTCRs and the radius of gyration of TCRs, Rg ratio (in **D**); both are computed using the pTCR model as a function of Lck* decay-length (nm) and depletion width between CD45 and TCR molecules (nm). **(E–G)** Bayesian networks representing probability density functions (PDFs) over variables from the surrogate KS model, Lck-A model, and pTCR model. In Bayesian networks, nodes represent random variables and directed edges represent potential statistical dependencies between a child node and its parents and conversely, statistical interdependencies between a child node and all other nodes given the values of its parents; edge direction does not indicate causality (37). Superscript in each variable’s name indicates the model associated with it. **(H–O)** Surrogate heatmaps showing the inferred mean **(H-K)** and standard-deviation **(L-O)** for the values of child variables in each surrogate model, given the values of their parents. The surrogate models are not yet coupled at this stage.

**Figure 6:**
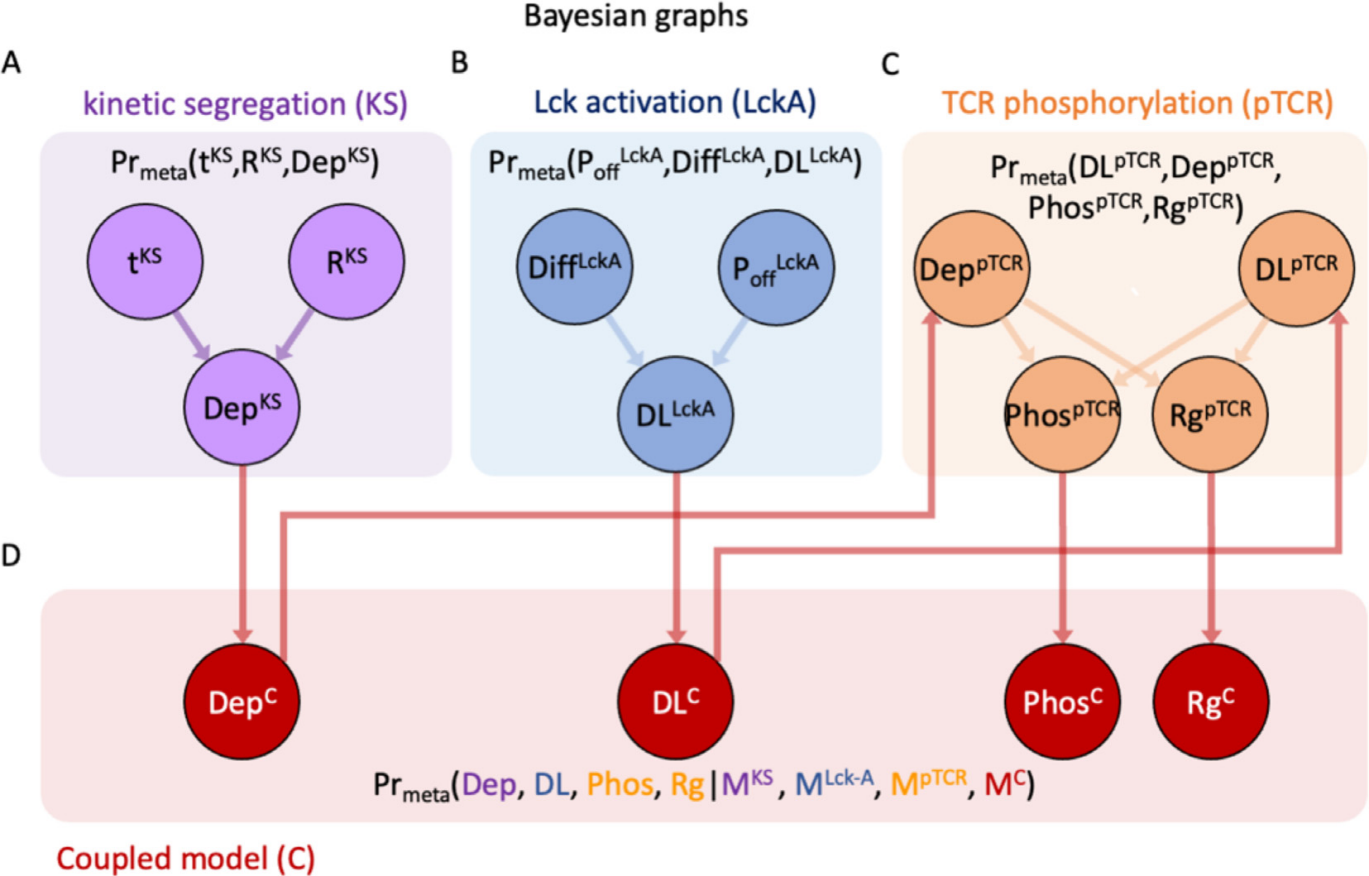
Bayesian metamodel of T-cell activation. This is a Bayesian network representing the joint probability density function (PDF) coupling variables from the three surrogate models. **(A–C)** The Bayesian networks for surrogate models as in **Figures 5E–G**, rewired to facilitate coupling via intermediate coupling variables; edges do not represent causality but rather potential statistical dependencies (37). **(D)** Coupling layer in the Bayesian metamodel, relating variables from the different partial models. In Bayesian metamodeling, coupling variables represent a hidden layer corresponding to the modeled ground truth, which is used as a common point of reference (26).

**Figure 7:**
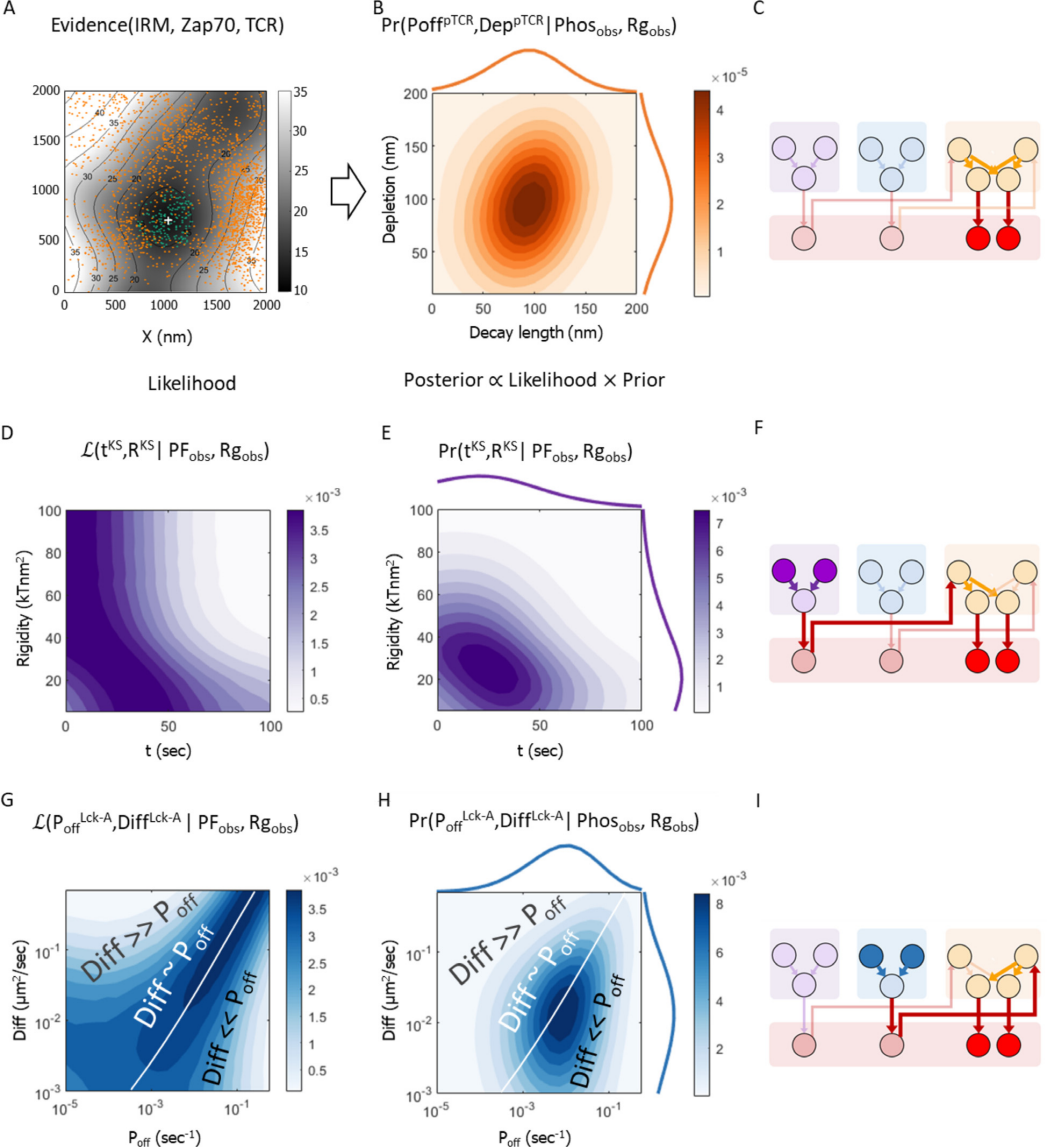
Metamodeling infers the probable parametric conditions for peripheral pTCR enrichment based on experimental data (as posterior). **(A)** Representative image used to compute input for metamodeling (**Supplementary Materials**, **Supplementary Figure S5**). Gray colormap with contours shows smoothed T cell topography measured by using Internal Reflection Microscopy (IRM) (23). Dark orange dots show measured locations of ZAP-70 molecules by using PALM (23). Green points are the TCR estimated distribution according to the membrane topography. Light orange points are the estimated locations of pTCR molecules (**Supplementary Materials**). The white cross indicates the center of mass of all the TCRs, phosphorylated and non-phosphorylated. **(B)** The posterior 2D probability density function (PDF) of the Lck* decay length (DL^pTCR^; x-axis) and TCR:CD45 depletion width (Dep^pTCR^; y-axis) variables of the pTCR model, given the empirical estimates of the phosphorylated fraction of TCR molecules (Phos^obs^) and the Rg ratio of phosphorylated vs. non-phosphorylated TCRs (Rg^obs^); the 1D histograms indicate the marginal PDFs for DL^pTCR^ (top) and Dep^pTCR^ (right) **(C)** Same Bayesian network as in **Figure 6** highlighting the variables associated with the conditional PDF in **(B)**; information backpropagates against the direction of the arrows. **(D, E)**. Likelihood **(D)** and posterior 2D PDF **(E)** of the time from activation onset (t^KS^; x-axis) and the rigidity (R^KS^; y-axis) variables in the KS model given the empirical estimates as in **(B)**. **(F)** Same as in **(C)** highlighting the variables associated with the process described in **(D, E)**. **(G–I)** Same as **(D–F)** for the Lck* deactivation rate (P_off_^Lck-A^; x-axis) and Lck diffusion coefficient (Diff^Lck-A^; y-axis) variables in the Lck-A model; the white line in **(G)** and **(H)** indicates a high-likelihood region where the values of the two parameters are proportional, in a way that is consistent with the observed peripheral nanoscale pattern of phosphorylated TCRs. Formally, the white line indicates the constraint Diff^Lck-A^=C·P_off_^Lck-A^, were C is a proportionality constant specified in units of μm^2^.

**Figure 8:**
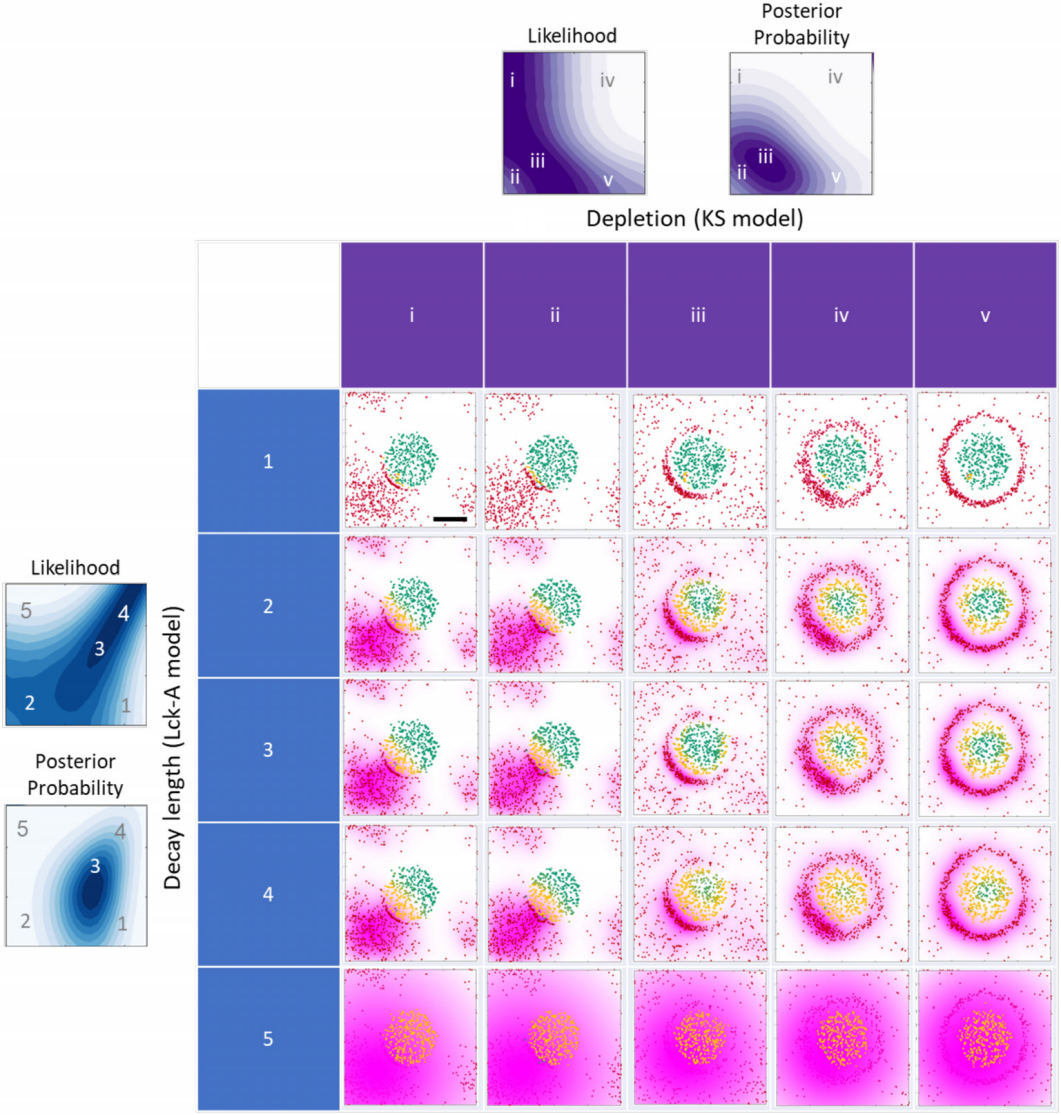
Graphical summary of the metamodel results and their implications. The grid displays the results of one Monte-Carlo simulation with the same initial conditions as in **Figure 2A**, middle row. The simulation results are plotted at three different times 5, 5, 25, 80, 80 sec at each column and marked by i-v respectively. For every row an Lck* distribution with increasing decay-length is plotted for values of 10, 80, 74, 100, 2000 nm and marked by 1-5 respectively. The two filled contour maps on the top and on the left are taken from **Figures 7D, E, G, H** respectively, where the letters and numbers indicate the coordinate (x, y, z) values in the 5x5 grid. Scale bar of 0.5 μm is shown in grid cell 1i.

The Results section is structured as follows: I. We present the gap between our current understanding of molecular mechanisms of T-cell early activation and empirical imaging data (**Figure 1**). II. We describe the details of how we construct the Bayesian metamodel of T-cell activation that combines three different molecular mechanisms (**Figures 2**–**6**; **Supplementary Figures S1**-**S3**; **Supplementary Tables S1**-**S9**). III. We use the resulting Bayesian metamodel to infer probable values for biophysical parameters of T-cell early activation given the empirical evidence (**Figure 7**; **Supplementary Figure S4**). IV. We discuss the implications of the propagated results on the specificity, sensitivity, robustness, and speed of T cell activation (**Figure 8**).

### TCR activation patterns are explained only through the combination of multiple microscopic mechanisms

We set out to model early T-cell activation during the first few tens of seconds from initial contact between a T cell and an APC. For simplicity, we focus on nanoscale patterns forming at a relatively restricted (2μm x 2μm) patch of the T cell plasma membrane and containing a single and representative membrane protrusion. The resulting patterns correspond to typical local clusters forming during early activation, and not the patterns that involve the whole contact area of the T cell at longer time scales (i.e. hundreds of seconds), where a full IS forms.

To quantitatively contrast the modeled patterns with empirical data, we use previously published single-molecule imaging of non-phosphorylated TCR, phosphorylated TCR (pTCR) and ZAP-70, and CD45, as well as the membrane topography of the early-forming IS (23). The pTCR molecules (observed via fixed cell imaging) and the ZAP-70 molecules (observed via live cell imaging) are proxies for local TCR activity, since phosphorylated TCR ITAMs recruit ZAP-70, thus leading to downstream signaling (24, 25). Note that the ZAP-70 molecules are not limited to the TCRs location since activated ZAP-70 can come off pTCR. This effect is nonetheless assumed to be local, as the ZAP-70 molecules remain at the plasma membrane and promote downstream signaling at neighboring microclusters (28).

We first studied whether the previously described kinetic segregation (KS) (13, 18–21) accurately captures key aspects of the observed molecular patterning. To this end, we employed InterCells, our Monte-Carlo simulation of the immune synapse implementing KS (21), to model (and to illustrate) the positions of membrane molecules and the topography of the plasma membrane throughout the process of cell activation (**Figures 1C**; only a membrane patch of an initial tight contact of radius < 0.5μm^2^ is shown). Briefly, InterCells balances the forces of the molecules, membrane elasticity, thermal fluctuations and reaction-diffusion stochasticity to account for the molecular positions and membrane topography (**Supplementary Figure S1**). We set the initial conditions of TCR and CD45 positions to resemble previous imaging results [e.g. Figure 2 in (19)]; these initial conditions are propagated by InterCells to yield the state of the simulated system over time, i.e. a trajectory of the plasma membrane topography and coordinates of related molecules.

As expected from our previous studies using InterCells (19, 21), its implementation of the KS model reproduced the empirical segregation patterns of CD45 from TCR upon formation of tight contacts (19, 20) (**Supplementary Figure S4**). However, the KS model could not explain the effect of this segregation on TCR phosphorylation, even when augmented with some straightforward assumptions about the TCR phosphorylation process – namely, an explicit representation of the Lck kinase (4), its activation by CD45 via dephosphorylation of its regulatory Y394 residue (22), its free diffusion across the plasma membrane (29), and the phosphorylation of the TCRs by the activated Lck (4). Under these assumptions, activated Lck (denoted Lck*) is distributed uniformly across the plasma membrane (**Figure 1C**, pink). For simplicity, we further assume that the phosphorylation of a TCR by Lck* is an all-or-none process rather than a gradual one, i.e. all ITAM tyrosines from a single TCR are either phosphorylated or dephosphorylated; and that the probability of a TCR to become phosphorylated is proportional to the density of the active Lck (i.e. Lck*) at the TCR location. Under these assumptions, the KS model predicts that the phosphorylation of the TCRs (esp. within the TCR clusters) is also uniform (**Figure 1C**, orange). Indeed, when plotting pTCR over the plasma membrane topography, simulated pTCRs cover the tightest contact area of the membrane uniformly (**Figure 1C**, center). However, this uniform distribution of pTCR in tight contacts appears to be inconsistent with recent experimental data (**Figure 1D**) (23). In other words, the KS model does not reproduce the empirical data under simple assumptions on Lck signaling.

We next examined whether somewhat less naive assumptions about Lck signaling could better support the empirical data. Nika et al. have shown that at the global cell level, ~40% of Lck molecules are primed or pre-activated in T cells, regardless of the activation state of the cells, being in “standby” for TCR activation (22). Thus, activation and deactivation processes need to balance overall Lck activity (30). For instance, Lck may be deactivated over time due to phosphorylation of the negative regulatory residue Y505 by activated kinases at the plasma membrane such as Csk (10, 31, 32), or dephosphorylation of the positive regulatory residue Y394 by protein tyrosine phosphatases such as SHP1, SHP2 (33), Lyp, PTPH1, PTP-MEG1, PTP-PEST (7, 34). The limited dwell time of Lck at the plasma membrane (~50 s) may further contribute to its time-dependent inability to phosphorylate ITAMs on TCR, and thus can be regarded as effective Lck* de-activation in our context (35). Thus, it is reasonable to make the following assumptions. First, we assume diffusing Lck molecules are deactivated at a certain rate following their activation by CD45. Second, if we further consider Lck’s activation by CD45 and diffusion across the plasma membrane (29), the distribution of activated Lck becomes locally non-uniform at the membrane, decaying exponentially as a function of the distance from CD45. Finally, the phosphorylation of TCR ITAMs by Lck arguably requires short-range contacts. Adding these simple assumptions on top of the basic KS model, our model now readily (yet only qualitatively) explains the observed non-uniform distribution of TCR phosphorylation, with a higher peripheral phosphorylation density adjacent to high local concentrations of CD45 (**Figure 1E**), and in qualitative agreement with the experimental data (**Figure 1D**).

In principle, the observed phosphorylation pattern of TCR during early activation at tight contacts can be thus described using a combination of three molecular mechanisms (**Figure 2**), each describing some aspect of its formation at the phenomenological level: (1) The mechanism of kinetic segregation (KS) of TCR and CD45 molecules (**Figure 2A**). (2) The mechanism for Lck activity and diffusion (Lck-A) describes the activation and deactivation of the mobile fraction of Lck molecules as they diffuse along the plasma membrane (**Figure 2B**). (3) The mechanism of TCR phosphorylation describes the phosphorylation of ITAMs by Lck from a short range (pTCR; **Figure 2C**). To conclude, the assumptions made in these three molecular mechanisms are jointly required and sufficient for qualitatively capturing the empirical evidence, that is, the enrichment of pTCR at the periphery of tight contacts (**Figure 1D**). II. Bayesian metamodeling of T-cell early activation.

To integrate the three molecular mechanisms of T-cell early activation, we employ our recent Bayesian metamodeling approach (26). Bayesian metamodeling provides a quantitative formalism for inferring how the individual mechanisms interact with one another, while explicitly accounting for the uncertainties associated with model parameter values and model predictions. Such uncertainties may arise from data and modeling limitations such as missing data, measurement noise, ambiguous assignments, and modeling simplifications. In addition, uncertainties may arise from the inherently stochastic nature of T-cell signaling. We construct the Bayesian metamodel in four steps: (1) construct quantitative partial models, each describing one of the abovementioned molecular mechanisms of TCR activation; (2) for each partial model, build a corresponding surrogate probabilistic model; (3) couple the surrogate models to each other in probabilistic space; and (4) backpropagate to update and harmonize the three partial models of the microscopic mechanisms. We now describe each of these steps in detail.

### Step 1: Construct a partial model for each molecular mechanism

We constructed three quantitative models, each describing one of the abovementioned molecular mechanisms involved in T-cell early activation. The models were implemented as follows (**Figure 2**; **Supplementary Tables S2**-**S9**; **Supplementary Materials**).

The kinetic segregation (KS) model was implemented by using InterCells, our previously described Monte-Carlo simulation of the immune synapse, which is already validated to predict the empirical kinetic segregation of TCRs and CD45 molecules (21). With InterCells, we calculated the dynamic spatiotemporal pattern of TCR and CD45 molecules on a T-cell plasma membrane during T-cell activation. Input to the model includes parameters such as membrane rigidity, repulsive and attractive interaction coefficients, and CD45 elasticity; as well as initial membrane configurations, TCR and CD45 locations on the T-cell membrane, and pMHC complexes on the APC membrane (**Figure 2A** left insets, **Supplementary Table S1**). The spatiotemporal configuration evolves over time (**Figure 2A**, insets). We focused on a squared patch of the plasma membrane with size of 2 *μm* x 2 *μm* with one cluster of TCRs at its center. The simulation takes into account forces between the molecules, membrane elasticity, thermal fluctuations, reaction-diffusion processes and membrane topography (**Supplementary Figure S1**). The output of the KS model is the spatial arrangement of TCR and CD45 molecules, from which we compute the width of the depletion zone (in units of nm). This zone is an area between the TCR and the CD45-enriched domains from which both types of molecules are apparently absent (**Supplementary Figures S2**, **S3**) (19).

The Lck activation (Lck-A) model was implemented using a random walk simulation. The simulation input includes a diffusion coefficient for the Lck/Lck* and an ‘off’ rate (*P_off_*), corresponding to the probability of an active Lck* molecule to become deactivated per second (**Figure 2B**). The simulations were initiated from a single CD45 molecule in the middle of a 2 μm x 2 μm grid, and 1000 Lck molecules at the center of the grid. During the simulations, mobile Lck molecules diffuse freely over the grid; a diffusing Lck molecule is activated upon encountering a CD45 molecule; and the activated Lck molecule (Lck*) diffuses and is deactivated with probability *P_off_* per second (**Figure 2B**, middle). While this deactivation occurs spontaneously in the model, it can occur, for instance, due to an encounter with deactivating kinases and phosphatases that were suggested to dephosphorylate Lck, such as Csk (10), SHP-1 (36), and SHP-2 (10), and treated implicitly by the model as spontaneous deactivation. We computed the two-dimensional coordinates of 1000 Lck* random walk paths that began at the grid center (**Figure 2B**, middle). The distances from the origin at the end of the simulation (after 1000 steps) are summarized in a radial histogram. From the histogram we compute the average decay length of Lck* (**Figure 2B**, right). The output of the Lck-A model is the magnitude of the decay-length in nm.

The TCR phosphorylation (pTCR) model was implemented by calculating the spatial distribution of the phosphorylated TCRs (pTCRs). The model takes the outputs of the KS model and the Lck-A model as input. Specifically, it includes the coordinates of the TCR and CD45 molecules from the KS model (**Figure 2C**, top left) and the decay-length parameter for Lck* from the Lck-A model (**Figure 2C**, bottom left). The Lck* distribution is implemented as a 2D radial probability density function relative to each CD45 molecule (**Figure 2C**, center bottom), summed over all the CD45 locations (**Figure 2C**, center top). The probability of a TCR to become phosphorylated is then assigned proportionally to the sum of all Lck* densities (**Figure 2C**, right top). CD45 molecules segregated from TCRs do not affect TCR activity directly, as they require direct contact with TCR for ITAM dephosphorylation. The output of the pTCR model is summarized by two variables that describe the distribution of the pTCR molecules relative to the distribution of all TCR molecules in the contact area (regardless of their phosphorylation state) (**Figure 2C**, bottom right). These two output variables are as follows: 1) *Phos*, the fraction of phosphorylated TCRs out of all TCRs (**Figure 2C**, bottom right, yellow and green horizontal arrows, resp.). 2) *Rg ratio*, the ratio between the root-mean-square deviations (RMSD) of phosphorylated TCRs from the center of mass of all TCRs, and the radius of gyration (Rg) of all TCRs (**Figure 2C**, bottom right, yellow and green vertical arrows, resp.). *Phos* quantifies the relative phosphorylation level in a cluster of TCRs, whereas *Rg ratio* quantifies the dispersion of pTCRs relative to a cluster of TCRs: it is smaller than 1 when pTCRs are concentrated near the center of all TCRs, it is equal to 1 when pTCRs are dispersed similarly to all TCRs, and it is greater than 1 when pTCRs are dispersed at the periphery of all TCRs.

#### Preliminary exploration of relation between variables in the three models

Before integrating the three models *via* metamodeling, we made a preliminary assessment of how the parameters of the various partial models jointly influence T-cell early activation. In other words, we assessed how the precise details of each of the three molecular mechanisms contribute to the shaping of the pattern of phosphorylated TCR molecules at the early IS (**Figure 3**). First, we mapped how key input parameters of each partial model determine the values of its output variables; for example, we assess how parameters like the plasma membrane rigidity affect the width of the depletion zone between TCR and CD45 molecules in the KS model, or how the deactivation rate of Lck* molecules affect their spread in the Lck model. Second, we identified output variables of the first two models (KS and Lck-A) that can be coupled to input parameters of the third model (pTCR); specifically, the spatial positions of TCR and CD45 molecules are outputs of the KS model, the spatial distribution of Lck* given the positions of CD45 molecules is an output of the Lck model; and together, these output variables inform input variables for the pTCR model.

Based on this relation between the three partial models, we can now assess how input parameters of one model influence output variables in a different model and obtain rough estimates for parameter values that are most consistent with empirical evidence. For example, we evaluate how plasma membrane rigidity in the KS model and the deactivation rate of Lck* molecules in the Lck-A model may affect the output phosphorylation pattern in the pTCR model (**Figure 3**). In the KS model, we evaluate how increasing the input value of the plasma membrane rigidity from 0 to 100 kTnm^2^ alters the output topography of the plasma membrane at t = 100 sec, and consequently, the output width of the depletion zone between CD45 and TCR molecules (**Figure 3A**). This effect of membrane rigidity is rationalized by the need to accommodate the height difference between CD45 and TCR molecules (~ 37 nm) at the interface with the APC (**Figure 1C**). When the input value of the rigidity parameter is small, the membrane can bend sharply and thus the ring of bulky CD45 molecules is closer to the cluster of TCR molecules, resulting in a narrow depletion zone. Conversely, as the rigidity increases, the membrane is bent more moderately, and the CD45 molecules are pushed to the periphery and away from the TCR molecules, with a wider depletion zone. In principle, these results can be contrasted with experimental imaging of TCR and CD45 segregation (19) to infer plausible values for the rigidity parameter (**Supplementary Figure S4**). As a negative control, we verified that there is no depletion zone, and in fact, no rearrangement of the CD45 and TCR molecules when the membrane rigidity parameter is set to zero (**Figures 3A, C**, bottom row). Similarly, in the Lck-A model, decreasing the value of the $P_{off}$ input parameter (i.e. the rate at which Lck-A is deactivated) increases the output decay-length of Lck* molecules relative to CD45, from 20 nm to 5000 nm (**Figure 3B**).

We next modified both the rigidity parameter from the KS model and the $P_{off}$ parameter from the Lck-A model jointly, resulting in different combinations of nanoscale patterns of TCR, CD45 and Lck*. The nanoscale patterns differ with regard to both the width of the depletion zone separating CD45 and TCR molecules computed in the KS model, and in the decay-length of Lck* molecules computed in the Lck-A model (**Figure 3C**). These outputs were then used as input parameters for the pTCR model, revealing how the input parameters from the KS model and Lck-A model influence the phosphorylation patterns of TCRs (**Figures 3D, E**). As a result, we were able to identify a subset of input parameters within specific limits that yielded phosphorylation patterns compatible with the observed data, qualitatively at first (**Figures 3D, E**, plots outlined in red). Notably, both the membrane rigidity and decay-length parameters needed to fall within certain thresholds to achieve the desired non-uniform phosphorylation of TCRs. Thus, we can roughly estimate how the input to the first two models determines the output of the third model; in other words, we estimate ranges of parameter values that are broadly compatible with empirical observations.

### Step 2: construction of three surrogate probabilistic models

To proceed with the integration of the three partial models *via* metamodeling, we next convert each of them into a corresponding surrogate probabilistic model. All surrogate models share the same representation and thus amenable to subsequent coupling, regardless of their original representation (26). Formally, a surrogate model is a probability density function (PDF) describing the statistical relations among some model variables. Here, we construct the surrogate probabilistic model for each of the partial models, namely the models of kinetic segregation (KS), Lck activation (Lck-A) and TCR phosphorylation (pTCR), as follows. First, we select a subset of the model’s variables that describe properties of interest, setting appropriate prior distributions for each variable. The prior distributions are based on published experimental results and theoretical information with wide confidence intervals (**Figure 4**). This approach allows substantial room for adjustments following subsequent coupling of the models and introduction of empirical evidence in later steps. Second, we systematically evaluate how changing some variables (e.g. free parameters, or model inputs) influences other variables (e.g. model outputs) in the subset (**Figures 5A–D**). Third, we construct a Bayesian network (37) describing a family of PDFs over the variables (**Figures 5E–G**). Finally, we infer the parameters of the Bayesian network that best approximate the observed joint distribution over all variables using No U-Turn Monte-Carlo and variational inference (38, 39) (**Figures 5H–O**, **Supplementary Materials**).

To construct a surrogate model for the KS model, we systematically mapped how the output depletion width (in nm) changes as a function of time (in sec), and plasma membrane rigidity (in kTnm^2^) (**Figure 5A**). We then learned the joint distribution over all three variables using an appropriate Bayesian network (**Figure 5E**); that is, a surrogate probabilistic model that reproduces the influence of time and plasma membrane rigidity on the output depletion width in the original KS model (**Figure 5H**) with the added benefit of rigorous quantitative estimates of the uncertainty in this output (**Figure 5L**). The model’s details are described in **Supplementary Materials** and in **Supplementary Tables S2**, **S3**. Similarly, we computed a surrogate model for the Lck-A model, representing a joint distribution over the output decay-length (in nm) and the input Lck* decay-probability rate, *P_off_* (in sec^-1^) and diffusion coefficient (in μm^2^/sec) (**Figures 4B**, **5B, F, I, M**; **Supplementary Materials**, **Supplementary Tables S4**, **S5**). Finally, we computed a surrogate model for the pTCR model, representing a joint distribution over two outputs of the pTCR model, phosphorylation (Phos.) fraction and radius-of-gyration (Rg) ratio, and two inputs, the decay-length of Lck* (in nm) and the depletion width between TCR and CD45 molecules (in nm) (**Figures 4C**, **5C, D, G, J, K, N, O**; **Supplementary Materials**, **Supplementary Tables S6**-**S9**).

Once we have the individual surrogate models (**Figures 4**, **5**) we integrate them into a metamodel where outputs of certain models are used as inputs to other models (**Figure 6**).

### Step 3: coupling of the three surrogate models in probabilistic space

To combine the three surrogate models into a single Bayesian metamodel, we use a Bayesian network to represent a joint PDF over their variables (**Figures 6A–C**), introducing four *coupling variables* to facilitate this coupling (**Figure 6D**). In Bayesian metamodeling, coupling variables are used as intermediates for formally describing the statistical relations between different models (26). Importantly, the arrows in a Bayesian network should not be interpreted as causal, rather they only represent potential conditional dependencies between a child node and its parents, and conversely, conditional independence between a child variable on all other nodes given the values of its parents (37).

### Step 4: backpropagation of updated variable PDFs

Using the joint PDF over the variables from all surrogate models, we can now infer the probability of variables from any model given the values of variables from any other model, as exemplified in the bottom equation of **Figure 6D**.

#### Using experimental evidence to infer biophysical parameters underlying early TCR activation

Having constructed a Bayesian metamodel that combined three molecular mechanisms of T-cell early activation, we can use it in the ‘reverse’ direction, updating the prior probabilities of parameters from some models (**Figure 4**) by backpropagating experimental data on variables from another model (**Figure 7**). Namely, we use metamodeling to identify the most probable parameters in all three models that together rationalize the observed nanoscale pattern of peripheral pTCR enrichment at T-cell early contacts, given empirical data on the IS. These data include experimental imaging of active TCR molecules and membrane topography from a previously published dataset [(23); **Supplementary Figure S5**], combined with theoretically inferred estimates of distributions of molecules that were not measured directly (**Figure 7A**). The experimental data includes the smoothed T cell topography measured using IRM (23), and locations of ZAP-70 molecules measured using PALM, serving as a proxy for phosphorylated ITAMs on TCRs (23). The theoretically inferred estimates include the distribution of TCR molecules, inferred from the observed membrane topography based on experimental measurements of the influence of the latter on the former (23).

In the following, we briefly describe the technical process by which we employ metamodeling to find the most probable set of parameter values given the empirical evidence via backpropagation. We backpropagate the empirical data with their uncertainties from the pTCR model to the inferred parameters in the KS and Lck-A models. First, we estimate the empirical fraction of phosphorylated TCRs out of all TCRs in a cluster of TCRs (*Phos_obs_* =22%) and the empirical Rg ratio of phosphorylated TCRs and non-phosphorylated TCRs relative to the center of the cluster of TCRs, *i.e.* the tendency of pTCRs to be localized in the periphery of the cluster (*Rg_obs_* =1.31). Second, we use these empirical estimates as observed values for the *Phos^C^* and *Rg^C^* coupling variables of the Bayesian metamodel, respectively (**Figures 6D**, **7C, F, I**, filled red circles). Third, we infer the posterior PDFs over parameter values in each of the three models (**Figures 7C, F, I**, filled orange, purple, and blue circles, resp.) given these observed values. In accordance with Bayes theorem, these posterior PDFs (e.g. **Figures 7E, H**) are proportional to the product of the corresponding data likelihoods (**Figures 7D, G**, resp.) and the prior probabilities (**Figures 4A, B**, resp.) of specific parameter values, where the data likelihood reflects the probability of the observed data given specific parameter values (**Figures 7D, G**); and the prior probabilities reflect our prior knowledge on acceptable parameter ranges (**Figure 4**).

Through the indirect probabilistic interpretation of experimental evidence, Bayesian metamodeling enables us to infer biophysical parameters that are recalcitrant to direct experimental measurements. For example, for the KS model, we use the metamodel to infer high likelihood estimates for *t^KS^*, the time following activation *R^KS^*, and the membrane rigidity parameter, given the empirical data (**Figure 7D**). These likelihood estimates are narrowed down when we also consider the prior estimates of these variables (**Figure 7E**). Similarly, for the Lck-A model, we infer the most likely (**Figure 7G**) and most probable (**Figure 7H**) values for the *Diff^Lck-A^*, the diffusion coefficient of Lck, and *P_off_^Lck-A^*, the off-rate of Lck*, given the empirical data. These parameters relate to the dynamics of a specific state of this molecule, and thus, they could not have been directly measured in the imaging experiments.

### Implications on T-cell activation

The metamodel indicates that to account for the observed nanoscale patterns, both the width of the TCR:CD45 depletion zone (KS model) and the decay length of active Lck (Lck-A model) are within a similar range of approximately 40-150 nm (**Figure 7B**). In turn, the TCR:CD45 depletion width depends on the values of the time following activation and rigidity parameter (**Figures 5H**, **7D–F**), and the activity range of Lck depends on a balance between the diffusion coefficient of Lck and the off-rate of Lck* (**Figures 5I**, **7G–I**). Further quantifying the latter dependency, we observed a scaling law, where the activity range of Lck* scales approximately with the inverse square root of *P_off_*/*Diff*, which also has units of length (**Supplementary Figure S8**). Thus, the activity range of Lck* can be easily modulated by changing either the de-activation rate of Lck* (7, 10, 31–34), its mobility (35, 40), or both.

This observation gives rise to three different regimes in the 2D likelihood function spanned by *Diff* and *P_off_* (**Figure 7G**). When the value of *Diff* is approximately equal to *C·P_off_*, where C is a proportionality constant specified in units of μm^2^ (**Figure 7G**, white line) the activity range of Lck* remains approximately constant (**Figure 5I**, diagonal contours), leading to high data likelihood when $Diff\approx C\cdot P_{off}^{\alpha }$ with an α value of approximately 1 (**Figures 7G**, **8**, rows 2-4). Indeed, theoretically, for a fixed activity range, we expect 2D diffusion to set the relation $Diff\approx C\cdot P_{off}^{\alpha }$ (41), since *P_off _* ~ 1/t and where α=1 corresponds to a normal diffusion. When the value of *Diff* is small relative to *C·P_off_*, Lck activity has a short range (**Figure 5I**, bottom-right), and as a result, nearby TCRs cannot be phosphorylated, inconsistently with the observed nanoscale pattern (**Figure 7G**, bottom-right region; **Figure 8A**, row 1). When the value of *Diff* is large relative to *C·P_off_*, Lck activity has a long range (**Figure 5I**, top-left), and as a result, all TCRs in the nearby cluster are phosphorylated, again inconsistently with the observed nanoscale pattern (**Figure 7G**, top-left region; **Figure 8**, row 5). Finally, the prior estimates of the model parameters further narrow down the range of probable parameter values for Diff and $P_{off}$ based on prior knowledge (**Figure 4**), resulting in their posterior estimates given the imaging data (**Figure 7H**).

We conclude by considering the implications of varying TCR depletion patterns and Lck activity ranges on TCR phosphorylation. For the TCR depletion patterns, we showed how the depletion width could change due to e.g. changes in biophysical parameters such as the membrane rigidity. The depletion width might be affected by other factors, such as differences in the length of different splicing isoforms of CD45 (20, 42). We simulated the patterning of TCR with such isoforms and found that an increase in the length of C45 resulted in a wider depletion zone (**Supplementary Figure S6**).

We consider three regimes of Lck activity ranges (**Figures 7G, H**). In the first regime, the values of Diff and $C\cdot P_{off}$ are balanced, and therefore, the activity range of Lck matches the TCR:CD45 depletion width. In this case, TCR phosphorylation occurs at the periphery of the TCR cluster (**Figure 8**, row 2-4, columns i-iii). In the second regime, the value of Diff is small relative to $C\cdot P_{off}$ , and therefore, the activity range of Lck is smaller than the TCR:CD45 depletion width, and the extent of TCR activation is reduced (**Figure 8**, row 1). Finally, in the third regime, the value of Diff is large relative to $C\cdot P_{off}$ and therefore, the activity range of Lck is larger than the TCR:CD45 depletion width, and the extent of TCR activation is highest (**Figure 8**, row 5, i-v).

The activity range of Lck might be limited by additional factors, such as inhibition by Csk molecules that are recruited to phosphorylated CD3 ϵ (31, 32). To test the possible effect of such additional mechanisms, we created an additional partial model where we explicitly represented Csk recruitment, using pTCRs as proxies for phosphorylated CD3 ϵ (**Supplementary Materials**, **Supplementary Figures S7A–F**). This fourth model of Csk recruitment was tested for a more limited range of parameter values compared with the other partial models and is not yet fully integrated into our Bayesian metamodel. Nonetheless, our preliminary analysis suggests that compared with the pTCR pattern without Csk (**Supplementary Figures S7A, B**), a peripheral density of Csk molecules forming at the edge of TCR clusters (**Supplementary Figure S7C**) could further refine the observed ring-like pattern of pTCRs (**Supplementary Figures S7D–F**) by further preventing diffusion of residual Lck* to the interior of the TCR molecules. Thus, Csk recruitment may provide robustness to erroneous T cell activation, possibly in conjunction with other molecular mechanisms such as tyrosine phosphatase activity (43) or limited dwell time of Lck at the plasma membrane (43).

### TCR phosphorylation dynamics at varying pMHC:TCR affinities

So far, we have assumed that the association between pMHC and TCRs during the early contact between the T cell and the APC persists throughout the simulation timescale (100 seconds), corresponding to wild-type or engineered TCRs with ligand interaction half-lives on the order of tens of seconds (44). However, the strength of the pMHC:TCR association may vary considerably in physiological settings (44, 45). We next assess how this association strength may influence the extent and dynamics of TCR phosphorylation for varying Lck activity ranges. To systematically map this relation, we conducted 900 independent simulations of the system under 180 different parameter combinations.

**Figure 9:**
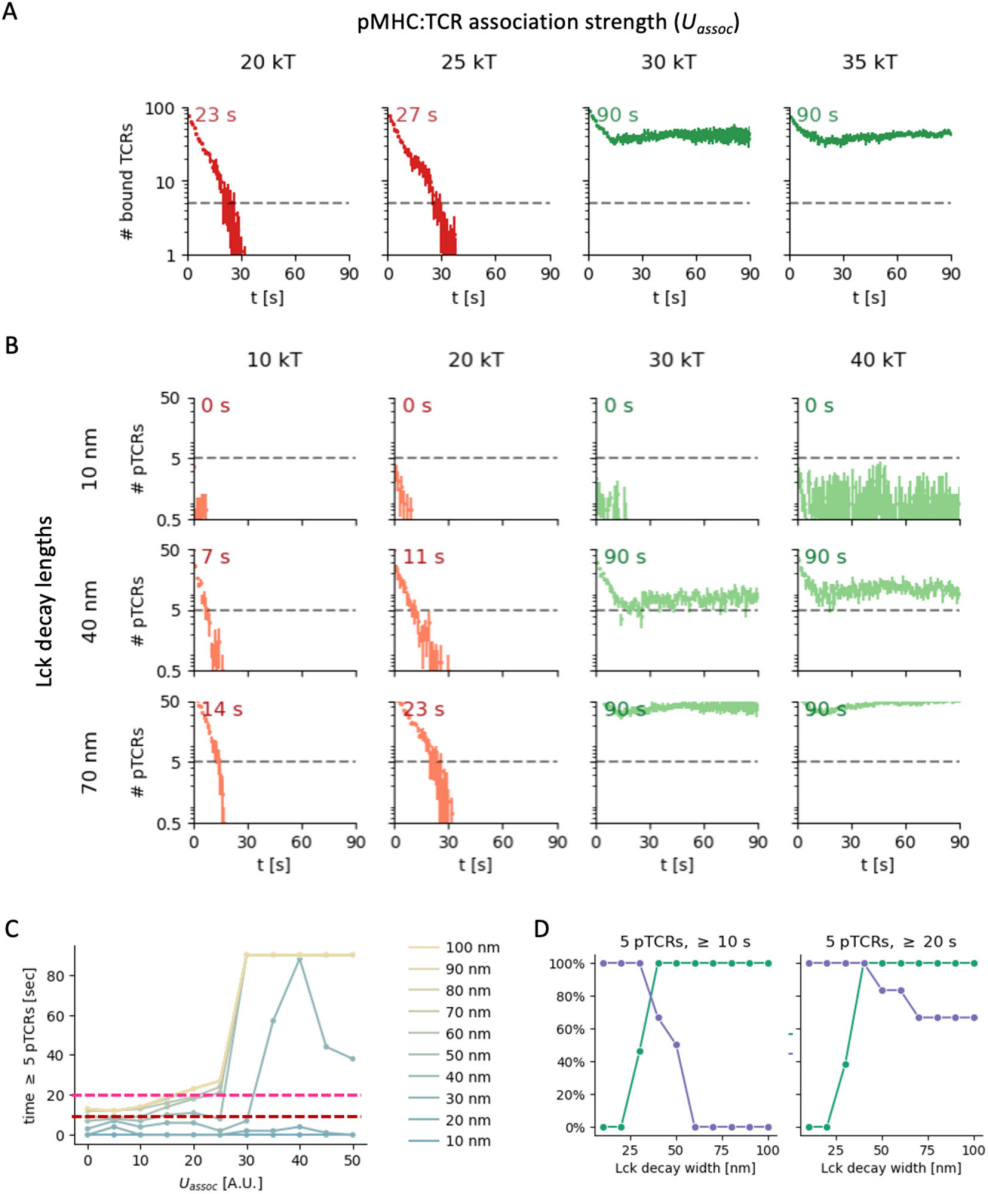
Effect of pTCR:MHC association strength on T cell signaling. **(A)** Number of bound TCRs as a function of time for different pMHC:TCR association strengths (columns). **(B)** Number of phosphorylated TCRs as a function of time for different pMHC:TCR association strengths (columns) and Lck decay lengths (rows). **(C)** Maximal time with more than 5 pTCRs as a function of association strength for different Lck decay lengths (blue to yellow lines). The red and pink horizontal dashed lines indicate stimulation time thresholds of 10 s and 20 s, respectively. **(D)** Sensitivity (green) and specificity (purple-blue) of early T cell activation in a model where the activation requires stimulations by at least 5 pTCRs for at least 10 s (left) or 20 s (right), corresponding to the red and magenta thresholds in B, respectively. Each point in the curves shown is computed for all pMHC:TCR association strengths under study (*U_assoc_ = 5 – 50 kT*), with a threshold of 25 kT for distinguishing low and high association strengths as hypothetical negative and positive ground-truth results, respectively.

Our simulations focused on a specific and simplified scenario where an initial contact has formed between the T cell and an APC, but in which the T cell has not yet triggered downstream signaling. This scenario is motivated by the reported formation of metastable contacts during the non-specific microvillar scanning of the APC surface (46). As a simplifying assumption, we posit that during initial contact formation, a cluster of TCR molecules on the T cell surface associated with identical pMHC molecules on the APC surface. We then allow the contact to evolve over time by applying all three partial models in our metamodel, similarly to our previous analyses (**Figures 2** **–** **8**). As before, we map the number of bound and phosphorylated TCRs as a function of the Lck activity range, but this time, over a range of pMHC:TCR associations strengths (**Figure 9**; **Supplementary Figures S9**, **S10**). Specifically, we adjust the association strength by modulating the tendency of TCR molecules to dissociate from pMHC molecules using an input parameter U_TCR-pMHC_, hereafter simply denoted as U, in our KS model (**Supplementary Table S1**; **Figure 2A**, top right, binding potential in green). To establish the initial contact, we constrained the membranes of the T cell and APC next to each other for a period of 10 seconds, at a distance that is sufficiently small to allow TCR molecules to bind and detach from pMHC molecules (**Supplementary Materials**). We then released the distance constraint and let the contacts evolve over 90 seconds.

We first monitored the number of TCR molecules that are bound to pMHC molecules as a function of time (**Figure 9A**, **Supplementary Figure 9**). Initially, the TCR molecules gradually dissociated from the pMHC molecules for all simulation parameter values. However, the dynamics and eventual state of the contact at the end of the simulations (*t* =90 sec) are strongly influenced by the pMHC:TCR value of $U_{\mathrm{assoc}}$ , the association strength parameter. We observe two distinct behaviors with a relatively sharp threshold at $U_{\mathrm{assoc}}∼25\;kT$ . For all simulations with $U_{\mathrm{assoc}}>25\;kT$ , the number of bound TCRs initially decreased somewhat over the first few seconds, but then stabilized at a relatively high level (dozens of bound TCRs; **Figure 9A** and **Supplementary Figure 9**, green curves). In contrast, for all simulations with $U_{\mathrm{assoc}}\leq 25\;kT$ , we observed complete detachment of the TCRs and the entire contact within approximately 30 seconds (**Figure 9A**; **Supplementary Figure S9**, red curves). In other words, for higher pMHC:TCR association strengths, the contact between the T cell and APC persisted throughout the simulation time, whereas for lower pMHC:TCR association strengths, the contacts are transient.

We next considered how these two distinct behaviors may influence TCR phosphorylation dynamics. In our metamodel, the level and duration of TCR phosphorylation is computed by integrating the three partial models (KS, Lck-A, and pTCR models; **Figures 2**, **5**). In particular, bound TCRs are phosphorylated if they coincide with high local density of Lck*, where the distribution of Lck* depends on the spatial localization of CD45 molecules and the Lck decay length (**Figures 2** **–** **7**). As such, we could monitor the level and duration of TCR phosphorylation as a function of Lck decay length, for different pMHC:TCR association strengths (**Figure 9B**; **Supplementary Figures S10**, **S11**).

In all simulations, the number of pTCRs increases as the Lck decay length increases, reaching the total number of bound TCRs for Lck decay lengths beyond 60-70 nm. In the simulations with $U_{\mathrm{assoc}}>25\;kT$ (**Figure 9B** and **Supplementary Figure 10**, green curves), the number of pTCRs still plateaued after a few seconds for any Lck decay length, but the final phosphorylation level scales strongly with the Lck decay length. For $U_{\mathrm{assoc}}\leq 25\;kT$ , the duration over which TCR phosphorylation persists increases with increasing Lck decay length, again reaching its maximum for Lck decay lengths beyond 60-70 nm (**Figure 9B**, red curves; **Supplementary Figure S10**, red curves).

### Potential effect on TCR-mediated activation

We next turn to the potential effect of TCR phosphorylation dynamics on TCR-mediated activation for different Lck decay lengths and pMHC:TCR association strengths. The phosphorylation of TCRs is needed for the recruitment of downstream effectors (30). Given sufficient stimulation time, even weakly-interacting (but not self) peptides were shown to lead to T cell activation (44). As such, we consider a condition for TCR-mediated activation, where the T cell is activated if a certain number of phosphorylated TCRs persist over a minimal duration. This condition serves as a simple proxy for the effective duration of pTCRs signaling. Using this hypothetical condition, we assessed the effect on Lck decay length on TCR-mediated activation.

For the case of high pMHC:TCR association strengths ( $U_{\mathrm{assoc}}>25\;kT$ ; **Figure 9B** and **Supplementary Figure S10**, green curves), the number of persistent pTCRs exceeds a certain threshold, e.g. of 5 pTCRs (dashed lines in **Figure 9B**) only when the Lck decay length surpasses 30 nm. In contrast, for Lck decay lengths below or equal to 30 nm, the number of pTCRs lies below the threshold. Under the proposed TCR-mediated activation condition, the T cell would not respond to high affinity pMHCs (that is, a false-negative response, as in anergy). In other words, the Lck decay length must exceed a certain value to guarantee persistent TCR phosphorylation when encountering high affinity pMHC ligands (**Figure 9C**, right-hand side). For the case of low pMHC: TCR association strengths ( $U_{\mathrm{assoc}}\leq 25\;kT$ ; **Figure 9B** and **Supplementary Figure S10**, red curves), the time duration over which the number of pTCRs crosses the set threshold shortens considerably if the Lck decay length is lower than 40-50 nm. In contrast, for higher Lck decay lengths, the large Lck activity range may give rise to a sufficiently high levels of pTCRs for a sufficient time duration (e.g. 10 or 20 seconds), leading to a risk of false activation of the T cell in response to low affinity pMHCs (as in autoimmune response to self-peptides).

Finally, we consider the potential implications on the sensitivity and specificity with which T cells discriminate between pMHCs with low *vs.* high association strengths. For each Lck length, we compute the sensitivity and specificity using a threshold of $U_{\mathrm{assoc}}=25\;kT$ as a hypothetical ground truth separating negatives from positives, and assuming the above TCR-mediated activation condition. Indeed, our results suggest that an optimal Lck activity range is achieved at intermediate values that balance the sensitivity and the specificity (**Figure 9D**). This result is robust to the precise threshold used for the minimal number of pTCRs or the duration over which they are phosphorylated (**Figure 9C**, red *vs.* magenta; **Supplementary Figure S11**). For example, for an activation threshold of 5 pTCRs for over 10 seconds, an Lck decay length of ~40 nm provides the most accurate response (**Figure 9D**, left).

We conclude that an intermediate Lck activity range may be optimal under the proposed conditions in the following sense. For transient contacts due to low pMHC:TCR association strengths, a corresponding transient increase in TCR phosphorylation levels peter out rapidly, thus reducing the risk for false activation. In contrast, for persistent contacts due to high pMHC:TCR association strengths, a sufficient number of TCRs remain phosphorylated non-transiently, thus facilitating proper TCR-mediated activation.

## Discussion

T cell activation is a complex process that incorporates multiple microscopic processes. While the processes are inherently stochastic, there seems to be a high level of spatiotemporal organization and orchestration of the events, giving rise to robust TCR-dependent signaling and cell activation. Here, we focused on the earliest events of TCR signaling. Specifically, we integrate multiple partial models to account for previously unexplained patterns of TCR activation in early T cell contacts. To this end, we employed Bayesian metamodeling, which can seamlessly integrate models of various representations, including those relating to distinct but related processes. We first showed that only the integration of multiple partial models can capture the previously observed phosphorylation pattern of TCR in the surroundings of initial tight contacts of the T cell at the immune synapse. We then confronted the model with microscopy data not used during its construction and used the metamodel to infer the most probable parameters of the individual models. These parameters could account for the empirically observed nanoscale patterns.

High resolution imaging of the early IS showed that T cell activation occurs at tight contacts (19, 20, 23). Fernandes et al. have proposed that the KS model in early contacts should produce a uniform activation pattern of TCR across the tight contacts (47). Still, imaging by Razvag et al. (23) has shown that TCR activation is enriched at the periphery of early and tight T cells contacts with activating coverslips. Thus, while the KS model successfully explains certain nanoscale patterns such as the formation of a depletion zone between TCRs and CD45 (44), it cannot explain other nanoscale patterns at the IS (**Figure 1D**). However, our metamodeling shows that the integration of the KS model with additional partial models (namely, the models of Lck activation and TCR phosphorylation) provides both sufficient and necessary conditions for capturing this patterning of TCR activation at the early contacts.

The interplay between CD45, Lck and pTCR molecules has been previously suggested to influence T cell activation (44). Still, the decay in the activity of Lck molecules over distance or time, and its effect on TCR phosphorylation, have not been sufficiently considered. In addition, the mobility of Lck is more complex than described in our model. Specifically, an immobile and self-inhibited fraction of Lck has been found in Lck clusters. Moreover, Lck recruitment to and within the IS can also be achieved by CD4/8 co-receptors, which we do not include in our current metamodel, but can be incorporated in future.

The pTCR ring-like patterning could persist more robustly during contact expansion through the involvement of additional surface molecules within the early T cell contacts. Specifically, the localization of the adhesion receptor LFA-1 in relation to the TCR and CD45 has been shown to correlate with intermediate contact tightness (45). LFA-1 molecules reside in between CD45 and TCR in the central supramolecular activation center (cSMAC) of the mature IS, and their co-engagement with their ligand result in enhanced TCR clustering, TCR segregation from CD45, and T cell activation (48). We recently showed that LFA-1 is enriched between TCR and CD45 also within the ‘depletion-zone’ in early forming T cell contacts using super-resolution microscopy, as also captured by InterCells (the KS model in the current manuscript (44). The conclusion from that study is that such patterning happens due to the incurred binding distance of LFA-1 and ICAM being in between the repulsing CD45 ectodomain, and the adhering TCR-pMHC interaction. Following these results, we conjecture that LFA-1 may stabilize the early (growing) contacts, and the associated segregation of TCR and CD45, and consequently, the pTCR ring-like patterning.

A key determinant of TCR phosphorylation and T cell activation is the association strength of TCRs with pMHC molecules on the surface of APCs (44). In the first part of this study, we focused on long-lived pMHC:TCR interactions, with relevance for some wild-type or engineered TCR constructs (43, 49), chimeric antigen receptors (CARs) (50), CD3-binding bispecific engagers (51) with long ligand interaction half-lives. However, the strength of the pMHC: TCR association may vary considerably under physiological conditions (51), resulting in much shorter interaction half-lives (52). In the second part of this study, we therefore studied the effect of pMHC:TCR association strength on the metamodel predictions.

In our metamodel, TCR phosphorylation is directly dependent on Lck decay length. To study the implications of Lck decay length on the extent and dynamics of TCR phosphorylation, we simulated a specific case where an initial contact between a T cell and an APC has formed, and through which a response of the T cell should be determined (**Figure 9**; **Supplementary Figures S9**-**S11**). As a proxy for TCR-mediated T cell activation, we employed a simple condition where T cell activation requires a certain number of phosphorylated TCRs to persist for a minimal duration of time. Under physiological conditions, low-affinity self-antigens are highly abundant on the surface of APCs. Therefore, cells contacts must be sufficiently mobile to efficiently scan the surface of APCs, but also sufficiently stable to distinguish foreign- and self-antigens. Thus, a tradeoff must be achieved for a contact dwell time, empirically estimated in the range of 3.5-6.0 sec for non-specific scanning (52, 53). Our results indicate that this ‘Goldilocks’ consideration may result in an optimal Lck activity range that is neither too long nor too short (**Figure 9D**). These findings (for this specific scenario) are insensitive to the specific choice of model parameters, including the thresholds for the minimal number and persistence time of pTCRs needed for activation (**Supplementary Figures S10**, **S11**).

Our metamodel assumptions admittedly simplify the complexity of TCR-mediated signaling under physiological conditions, and our results should be restricted to the simulated scenario. For example, aside from the association strength, the abundance of pMHC on the surface of APCs strongly affects the extent of TCR phosphorylation and T cell stimulation (53). Moreover, in some cases, even repeated short-lived interactions may lead to T cell triggering, including at single pMHC sensitivity (54). Such parameters can be mapped using our metamodel of early T-cell signaling along with additional scenarios of T cell interactions with APCs. The metamodel also assumes that unbound TCRs cannot be phosphorylated by Lck*. This assumption is consistent with the reported release of intracellular CD3ϵ chains from the plasma membrane following pMHC:TCR binding, thus making them available for phosphorylation (55, 56); and other ligand-induced conformational changes to the TCR ζ chains (57). It is also consistent with the need for a ‘safety’ mechanism given that ~40% of Lck molecules in the T cell are constantly active (47, 57). Other studies have emphasized a primary role for the exclusion of the membrane phosphatases from TCRs (47, 58). While these mechanisms are not mutually exclusive (58, 59), their roles could be further studied using our metamodel. Specifically, the pMHC-binding condition can be readily modified and its implications further evaluated, along with additional molecular mechanisms to prevent non-specific TCR phosphorylation and T cell activation.

The presented Bayesian metamodeling approach integrates three specific partial models of nanoscale patterning at the early-forming IS. This approach is scalable by design, allowing for its expansion using a broad range of existing computational models (59–64). Examples include minimalist models focusing on IS molecular patterns and phenomenology under various conditions, using finite difference schemes for numerical solutions (61); a reaction-diffusion model exploring molecular segregation driven by bond length differences (61); a thermal equilibrium model detailing cell-cell interactions and informing the interplay between cell wall elasticity, intercellular repulsion, and bond formation (65–67); a statistical-mechanical model capturing thermal fluctuations and spatial patterns (63); and a Monte-Carlo model analyzing phosphorylation-dephosphorylation signaling based on internal spin states (64); models of the interplay of on-off kinetics in TCR-pMHC interactions and specific T cell activation patterns within the immune synapse (47, 65); and consideration of the role of actin (66–68). While each of these models provides valuable insights on some key aspects of the evolving IS, arguably - none encompasses all microscopic mechanisms needed to explain the observed ring of phosphorylated TCRs. Moreover, traditional computational models often lack scalability in the sense that their systematic expansion is challenging. Bayesian metamodeling facilitates the integration of these models as partial components, progressively enhancing detail and completeness in modeling the early-forming IS, thereby capturing a growing number of IS features and functional cell outcomes in health and disease.

The current Bayesian metamodel focuses on early contacts made by engaged T cells with APCs. At later stages of contact evolution and immune synapse maturation, we expect additional molecular and cellular processes that are not incorporated into our model to come into play. Such mechanisms may include actin remodeling, protein transport, reorientation of subcellular organelles, protein recycling and more. Thus, our current model is still limited in its ability to capture molecular organization in the mature IS, especially the concentric bull’s eye pattern of the (c, d, and p)-SMACs (69). Still, the scalable properties of Bayesian metamodeling allow future incorporation of such molecular and cellular processes, towards a more comprehensive account of the more mature IS as well as additional T cell activation responses and functions.

More broadly, spatiotemporal organization of molecules determines the rates of local interactions. Thus, such interactions are a common regulation mechanism for a wide range of cellular processes, including cell sensing, signaling pathways, metabolic networks, transcription, translation, and more. Spatiotemporal dependencies and stochasticity (e.g. of molecular diffusion, interactions, etc.) directly translate to system complexity. This complexity becomes daunting, esp. when one tries to gain fundamental understanding of the system working. As such, it motivates approaches that attempt to describe complex processes, even at the whole-cell level, as a collection of submodules that describe various system functionalities jointly. Metamodeling has a unique and natural capability to describe complex systems in a tractable and scalable fashion (26). Thus, while our metamodeling approach was applied here to a concrete example of TCR activation, it could naturally grow to account for additional aspects of T cell signaling and functions, as well as additional cellular systems.

## Data Availability

The original contributions presented in the study are included in the article/**Supplementary Materials**, further inquiries can be directed to the corresponding author/s. The code for all models and metamodels can be found in the GitHub repository (https://github.com/ravehlab/immune-synapse-metamodeling), divided into subfolders for InputModels, SurrogateModels, and CoupledModels.
