# Supplementary material for: Bayesian metamodeling of early T-cell antigen receptor signaling accounts for its nanoscale activation patterns
Source: Front Immunol. 2024 Oct 25;15:1412221. doi: 10.3389/fimmu.2024.1412221 (PMC11543436; doi:10.3389/fimmu.2024.1412221)
Supplement: Supplementary file 1 [file DataSheet1.pdf]

Supplementary Methods: Bayesian metamodeling of early T-cell antigen receptor signaling accounts for its nanoscale activation patterns

Yair Neve-Oz[1,2], Eilon Sherman[1]*, Barak Raveh[2]*

[1]Racah Institute of Physics, The Hebrew University, Jerusalem, Israel, 91904
[2]School of Computer Science and Engineering, The Hebrew University, Jerusalem, Israel, 91904
* corresponding authors

Keywords: T cell, immunological synapse, Bayesian metamodeling, T-cell activation, kinetic segregation, LCK, T-cell receptor, CD45, peptide-MHC, super-resolution microscopy

Supplementary Methods

Models

In this section, we present the partial models used as input for metamodeling, including their conversion to probabilistic surrogate models and coupling. We specify the free parameters, input and output variables for each input partial model. All models were implemented in MATLAB (R2020b) or Python (3.7.6). The surrogate models were implemented using Python (version 3.7.6). The code for all models and metamodels can be found in the GitHub repository (https://github.com/ravehlab/immune-synapse-metamodeling), divided into subfolders for InputModels, SurrogateModels, and CoupledModels.

Surrogate models

Surrogate probabilistic graphical models. We created a probabilistic graphical model, specifically a Bayesian network [28] describing statistical relations among the membrane rigidity parameter κ, the time *t*, the depletion zone width.

Based on the output heatmaps we create a surrogate model. In the surrogate model we made an approximation of an output heatmap by using a two-dimensional surface $Z(t,\kappa)$ that is a function of random variables (RV's) (**Supp. Table S2A**). To run the Bayesian network, we used the PyMC3 (1) which is a probabilistic programming package for Python that allows users to fit Bayesian models using a variety of numerical methods, most notably Markov chain Monte Carlo (MCMC) and variational inference. We followed the following process:

1) Form an equation that can describe the characteristics of the output heatmap.
2) Choose random variables that can describe the heatmap correctly with wide enough margins. We use the observed t and κ as input.
3) Run the PyMC3 package to make a 'sanity check' of the results and see if the resulting random variable can fit the data.
4) If the result passes the 'sanity check' we can refer to it as a surrogate model. We train the surrogate model by running the model with the learned random variable over a batch of t and κ values that we choose. The resulting trained model is independent of the data.

We used PyMC3 v3.9.3 with a No-U-Turn (NUTS) sampler (2) and 4 Markov chains of 2000 steps. To create the trained model, we ran the model over a batch of t and κ. Time, t, was from 0 to 100 sec with 10 sec intervals. Plasma membrane rigidity, κ, was from 10 kT/nm^2 to 100 kT/nm^2 with 10 kT/nm^2 intervals. For details see **Supp. Table S2A**.

Kinetic segregation model

Input & Output: The Kinetic Segregation (KS) model calculates the spatiotemporal pattern of TCR and CD45 molecules on a T-cell plasma membrane during T-cell activation. Input to the model includes model parameters and initial membrane configurations, TCR/CD45

molecules on the T-cell membrane, and pMHC complexes on the APC membrane (**Supp. Table S1**). The output of the model is the spatiotemporal trajectory of the model's configuration, as follows.

Model Interactions: The model assumes local equilibrium and describes interactions using Hamiltonian H over the model configuration (c). Initial conditions of the simulations: N_{TCR} ~ 500, N_{CD45} ~ 500 (**Supp. Fig. S1**). The inter-membranes distance, Δz = 70nm (white color) except at the locations of the molecules, where for the CD45 locations Δz = 50nm (resting length of CD45 molecule) and for the TCR locations Δz = 13nm (length of bound pMHC-TCR). pMHC molecules (not shown here) are uniformly scattered over the APC membrane with surface density of 300/μm^2. In the current study, the pMHC molecules are fixed and their density is constant, but in principle, the model allows pMHC molecules to diffuse and their spatial distribution and copy number can be modified. Molecules distribution and topography after 10,000 iterations (100 sec). The color bar represents inter-membranes distance.

$$H(c) = H_{membrane}(c) + H_{compression,CD45}(c) + H_{compression,TCR}(c) + H_{bind}(c).$$

$H_{membrane}$ is the total bending energy of the T cell and APC membranes due to changes in $\Delta z_{i,j}$, $H_{compression,CD45}$ and $H_{compression,TCR}$ are the spring energies associated with compressing the vertical length of the CD45 and TCR molecules, respectively, and H_{bind} is the binding energy of a molecule to a molecule on the opposite membrane.

$$H_{i,j\ membrane} = \frac{\kappa}{2a^2}\left(4\Delta z_{i,j} - (\Delta z_{i+1,j} + \Delta z_{i-1,j} + \Delta z_{i,j+1} + \Delta z_{i,j-1})\right)^2.$$

a^2 is the area of one square, κ is the membrane bending rigidity.

$$H_{compression,CD45} = \frac{k}{2}(h_{CD45} - h_{0,CD45})^2, if\ h_{CD45} < h_{0,CD45}, otherwise\ H_{compression,CD45} = 0.$$

and

$$H_{compression,TCR} = \frac{k}{2}(h_{TCR} - h_{0,TCR})^2, if\ h_{TCR} < h_{0,TCR}\ , otherwise\ H_{compression,TCR} = 0.$$

k is the spring constant of the molecule.

$$H_{bind,CD45} = u_{bind,CD45}\ , if\ \Delta z_{i,j}\ is\ in\ the\ molecule\ binding\ range.$$

$$H_{bind,TCR} = u_{bind,TCR-pMHC}\ , if\ TCR\ and\ pMHC\ are\ in\ the\ same\ square.$$

Planar interactions: The planar interactions of the molecules are implemented in the property that two molecules on the same membrane cannot be at the same square at the same time (interacting like hard spheres).

Vertical molecules interactions: Receptor-ligand interactions occur when a TCR and a peptide-MHC have the same planar location. In this case they are forced to be bound and at that location

the inter-membranes distance, Δz, will be 13 nm. Molecule-membrane interactions: CD45 molecules interact with the APC membrane as repulsive springs when Δz < CD45 resting length.

Model dynamics. We evaluate the model dynamics using Reaction-diffusion Markov-Chain Monte-Carlo (3,4). A typical simulation runs for 10,000 iterations. To simulate Brownian diffusion of each molecule along the membrane, we sample a random molecular step r in a random direction (uniformly sampled between 0 and 2π radians), and with a magnitude that is the absolute value of a normally distributed scalar with mean 0.0 and standard-deviation $\sqrt{4 \cdot D \cdot \Delta t}$ nm, approximating the normally-distributed function along the x-axis and y-axis of standard-deviation $\sqrt{2 \cdot D \cdot \Delta t}$ nm; D is the diffusion coefficient in units of $nm^2 \cdot s^{-1}$. Therefore, we can treat the propagation of the simulation as propagation in time with time step = Δt, which was set to 0.01 s unless stated otherwise. We used periodic boundary conditions (molecules that exit at one side enter on the other side). At every iteration, all the TCR and CD45 molecules are moved to a new location with the random step r; the move is accepted or rejected using the Metropolis criterion: it is accepted with $P_{accept} = 1$ if the energy at the new state H_{new} is lower than the energy at the old state H_{old}. If the energy at the new state is higher, the attempt is accepted with $P_{accept} = e^{\Delta H}, where\ \Delta H = H_{new} - H_{old}$.

If a molecule attempts to move into a new square that was occupied in the previous iteration it is rejected. If more than one molecule attempts to move into the same new square all these attempts are rejected. The molecules that their lateral attempt was accepted change their height, h, to the new $\Delta z_{i,j}$ at their new locations and are accepted or rejected according to the Metropolis criterion for molecules. In addition, the inter-membranes distance $\Delta z_{i,j}$ at every grid square is changed by a normally distributed random step dz with mean = 0 and standard deviation = 1nm.

Membrane dynamics: at all membrane coordinates, the membrane attempts to move up or down to a new $\Delta z_{i,j}$. The $\Delta z_{i,j}$ of a membrane coordinate i,j that is occupied by a TCR and a pMHC is constrained to have $\Delta z = h_{0,TCR}$. The rest of the new $\Delta z_{i,j}$ are accepted or rejected according to the Metropolis criterion for membranes. Therefore, in this study, bound TCRs are effectively immobile (since the pMHCs are immobile). Unbound TCRs may diffuse.

Supplementary Table S1: Parameters of the KS model

Name	Description	Default value	Units	References
Array size	size width x size length.	2000x2000	nm	
a	Area unit that divides the array to square units that has approximately the lateral size of a molecule.	10	nm	WL, InterCells
Δt	Iteration length.	0.01	sec	InterCells

N_{iter}	Number of iterations	10,000	-	InterCells
$h_{0,TCR}$	Vertical resting length of the TCR.	13	nm	WL
$h_{0,CD45}$	Vertical resting length of the CD45.	50	nm	WL
N_{TCR}	Total number of TCR molecules.	~125	–	
N_{CD45}	Total number of CD45 molecules.	~500	–	
D_{TCR}	Diffusion coefficient of TCR molecules.	10,000	nm^2/sec	WL
D_{CD45}	Diffusion coefficient of CD45 molecules.	11,000	nm^2/sec	WL
κ (kappa)	Membrane rigidity.	25	$kT \cdot nm^2$	WL
k	Spring constant	10 κ/a^2	kT/nm^2	WL
u_{CD45}	Binding energy of CD45 to APC membrane	-10	kT	
u_{TCR}	Binding energy of TCR to APC membrane	-10	kT	
U_{assoc}	Binding energy of TCR to pMHC	Effectively -inf during the simulaiton (k_{off} > 120 *sec*)	kT	
$P_{on,TCR}$	Probability of self-clustering of TCR when in contact with another TCR	0.995	sec^{-1}	InterCells
Input parameters (initial configuration):				

TCR initial distribution	Circle with uniformly distributed molecules.	Center = (1000,1000)<br>R = 400	nm	
CD45 initial distribution	Circle with uniformly distributed molecules.	Center = (400,400)<br>R = 400	nm	
Δz_0	Initial membrane height. It is constant everywhere except at the locations of the molecules.	70	nm	InterCells
$\Delta z_{0,TCR}$	Initial membrane height at the locations of TCR molecules.	$h_{0,TCR}$	nm	InterCells
$\Delta z_{0,CD45}$	Initial membrane height at the locations of CD45 molecules.	$h_{0,CD45}$	nm	InterCells

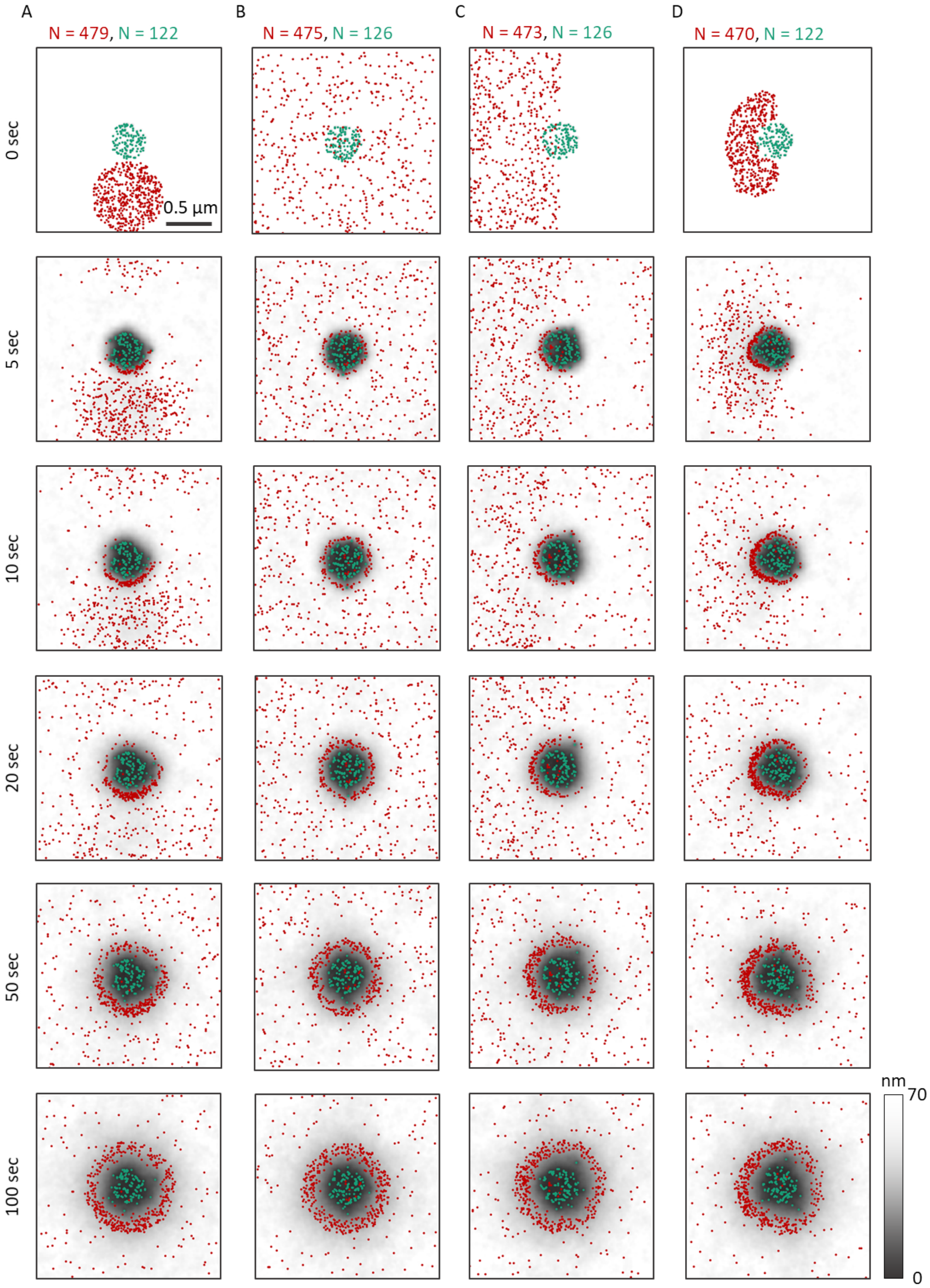

Supplementary Figure S1. **Monte-Carlo simulation with different initial conditions.** Simulation results using different initial conditions. The top row shows the initial conditions (t = 0) of four different simulations. The lower rows show the progress over time of the simulations. Despite the different initial conditions, the final state of the simulations (t = 100sec) is very similar.

Pair correlation function

To analyze the distribution of points in our simulations, we employed the pair correlation function (**Supp. Fig. S2**), which is defined as: $g(r) = \sum_{i} \rho_i(r)/\rho_0$. Here, $\rho_0 = N/A$, is the overall density (N is total number of points and A is total area). $\rho_i(r) = n_i(r)/a(r)$. (n_i is the number of points on a ring with radius r centered at point i. $a(r)$ is the area of a ring with radius r and width Δr and is set as the number of gray squares that makes the ring. We used the squares of the grid to form 'rings' that are consisted of squares within $r - \Delta r/2$ and $r + \Delta r/2$. Where $\Delta r = a$ (square size). To characterize the point distribution patterns, we computed both the univariate pair correlation function $g_{11}(r)$ and the bivariate pair correlation function $g_{12}(r)$ and characterized each g(r) curve by a single value. We demonstrate the process using **Supp. Fig. S2**: the overall area of the array is 49 squares; the total number of green points is 4 and the total number of red points is 6. Therefore, when calculating $g_{11}(r)$ and $g_{12}(r)$ where the circles are centered around the green point (green=1, red=2), ρ_0 = 4/49. For r = 1 (**Supp. Fig. S2A**), the area of the ring is 8 squares, there are 0 green points and 0 red points on the ring. Hence, $\rho_{i1}(1)$ = 0 and $\rho_{i2}(1)$ = 0. For r = 2 (**Supp. Fig. S2B**), the area of the ring is 12 squares, there are 2 green points and 2 red points on the ring. Hence, $\rho_{i1}(2)$ = 2/12 and $\rho_{i2}(2)$ = 2/12. For r = 3 (**Supp. Fig. S2C**), the area of the ring is 16 squares, there is 1 green point and 4 red points on the ring. Hence, $\rho_{i1}(3)$ = 1/16 and $\rho_{i2}(3)$ = 4/16. g(r) for A, B and C will be the sum of $\rho_i(r)$ when centering the circles at every green point and dividing by the total density of green points ρ_0.

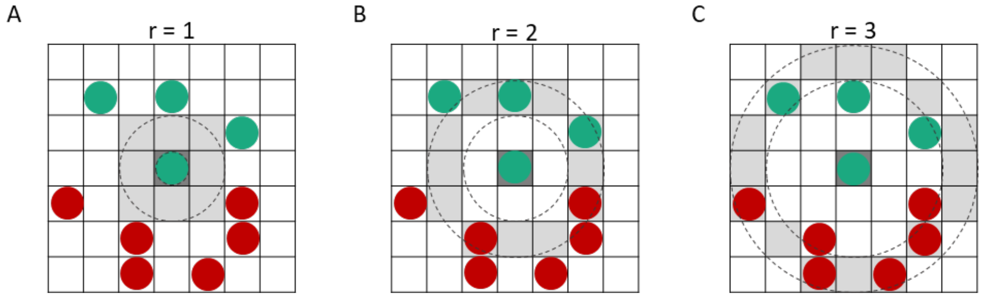

Supplementary Figure S2. **Pair correlation function method.** Computing g(r). $g(r) = \sum_{i} \rho_i(r)/\rho_0$. $\rho_0 = N/A$ is the overall density. $\rho_i(r) = n_i(r)/a(r)$, where n_i is the number of points on a ring with radius r centered at point i. $a(r)$ is the area of a ring with radius r and width Δr. We used the squares of the grid to form 'rings'. The overall area of the array is 49 squares, the total number of green points is 4 and the total number of red points is 6. Therefore, when calculating $g_{12}(r)$, where the circles are centered around the green points ρ_0 = 4/49. **A**. For r= 1, the area of the ring is 8 squares, there are 0 green points and 0 red points on the ring, therefore, $\rho_{i1}(1)$ = 0 and $\rho_{i2}(1)$ = 0. **B**. For r = 2, the area of the ring is 12 squares, there are 2 green points and 2 red points on the ring, therefore, $\rho_{i1}(2)$ = 2/12 and $\rho_{i2}(2)$ = 2/12. **C**. For r = 3, the area of the ring is 16 squares, there is 1 green point and 4 red points on the ring, therefore, $\rho_{i1}(3)$ = 1/16 and $\rho_{i2}(3)$ = 4/16. g(r) for **A**, **B** and **C** will be the sum of $\rho_i(r)$ when centering the circles at every green point and dividing by the total density of green points ρ_0.

To get the depletion distance from the bivariate pair-correlation function $g_{12}(r)$ we measured the distance (blue double edge arrow) from r = 0 to the first point of intersection of the

g(r) curve with the horizontal line of g(r) = 1. Here we show, at different times (0, 5, 10, 20, 50, 100 sec), the point pattern (inset) and the corresponding g(r) curve.

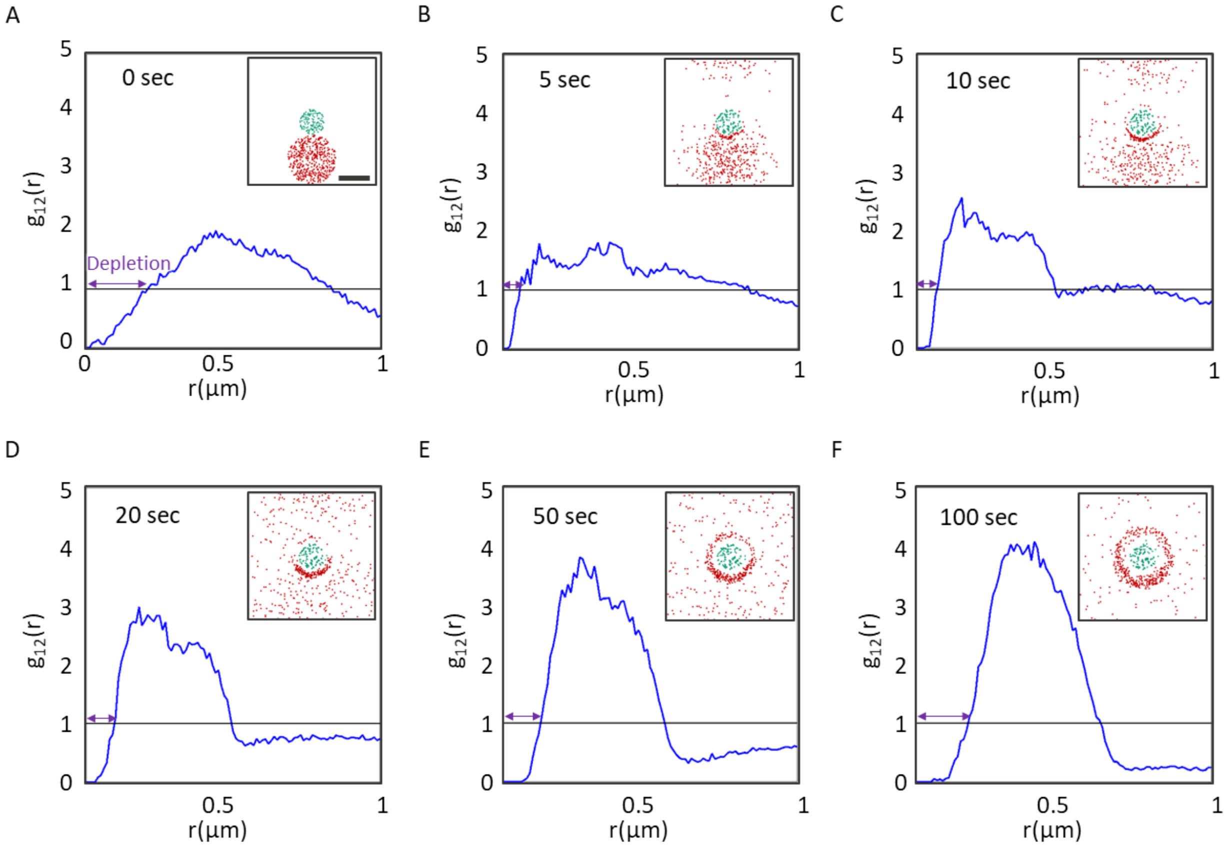

Supplementary Figure S3. Calculating the depletion size between TCR and CD45 molecules. The point pattern (inset) and the corresponding g(r) curve at different times (0, 5, 10, 20, 50, 100 sec). The depletion (purple double edge arrow) is the distance from r = 0 to the first point of intersection of the g(r) curve with the horizontal black line of g(r) = 1.

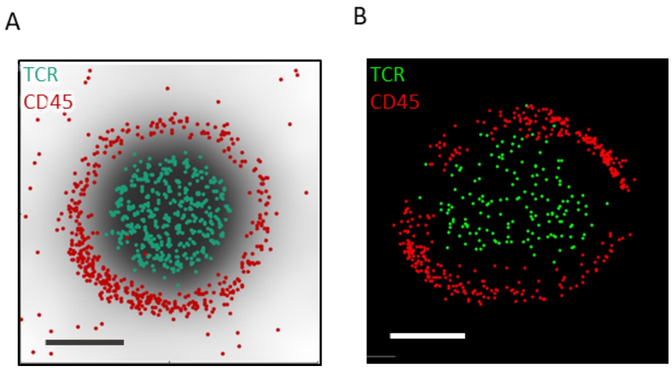

Supplementary Figure S4. Comparison between simulation setup and experimental results for the KS model (5). Point patterns of TCR and CD45 at an early contact of a T cell with an activating surface. **A.** Simulated pattern using Intercells. **B.** Sample experimental data captured using PALM. Scale bars are 0.5µm. An extensive experimental validation of the KS model has been discussed in an earlier study (5).

The surrogate KS model

Model input: To learn a surrogate model for the KS model, we first mapped its outputs for different input parameter values (**Fig. 2A**). We then created a probabilistic graphical model (6) that describes statistical relations among its variables in parameterized form (**Fig. 4A,E,H,L, Supp. Tables S2,3**); next, we fitted the probabilistic graphical model to recapitulate the statistical relations between the model parameters and its values (**Supp. Table S2**). The learned parameters of the KS model are shown in **Supp. Table S3**.

Kinetic segregation (KS) model

Variable type	Name	Description	Distribution type	Distribution parameters	Units
Free parameter	t^{KS}	Time	Uniform	$lower = 0$, $upper = 100$	sec
Free parameter	R^{KS}	Plasma membrane rigidity	Uniform	$lower = 0$, $upper = 100$	$kT \cdot nm^2$
Random variable	Dep^{KS}_{tScale}	Sigmoid height for t parameter	Normal	$\mu = 130$, $\sigma = 20$	nm
Random variable	Dep^{KS}_{tCen}	Sigmoid center for t parameter	Normal	$\mu = 50$, $\sigma = 10$	
Random variable	Dep^{KS}_{tDev}	Sigmoid width for t parameter	Normal	$\mu = 20$, $\sigma = 10$	
Random variable	Dep^{KS}_{RScale}	Sigmoid height for κ parameter	Normal	$\mu = 100$, $\sigma = 20$	
Random variable	Dep^{KS}_{RCen}	Sigmoid center for κ parameter	Normal	$\mu = 20$, $\sigma = 10$	
Random variable	Dep^{KS}_{RDev}	Sigmoid width for κ parameter	Normal	$\mu = 30$, $\sigma = 10$	
Random variable	Dep^{KS}_{output}	Output	Normal	$\mu = \frac{tScale}{1 + e^{-\frac{t-tCen}{tDev}}} + \frac{\kappa Scale}{1 + e^{-\frac{(\kappa-\kappa Cen)}{\kappa Dev}}}$, $\sigma = 20$	

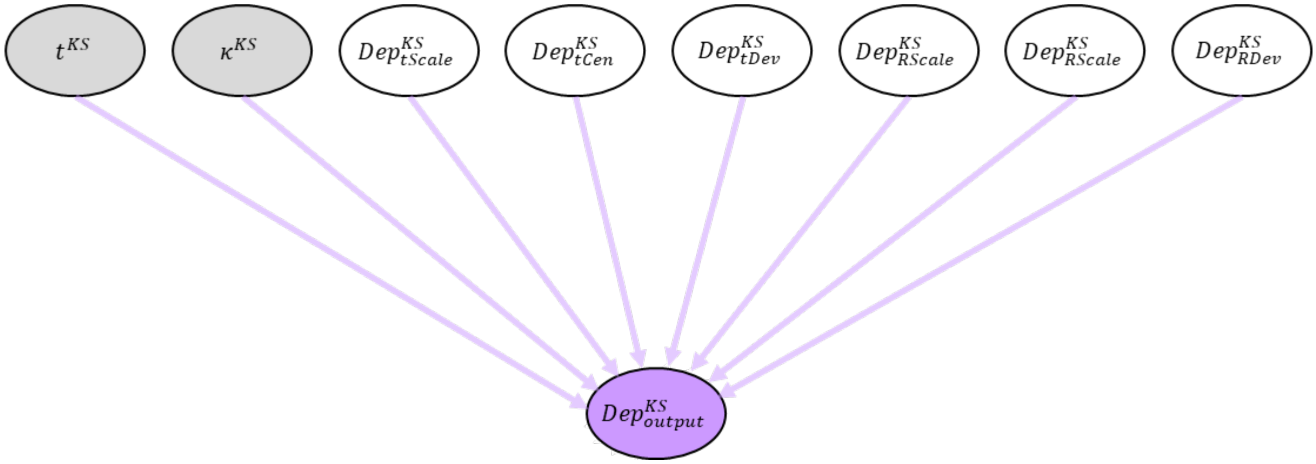

Supplementary Table S2 Random variables for the surrogate KS model before training. top: random variables for the surrogate KS model before training. **Bottom**: Bayesian network for the surrogate KS model before training.

Kinetic segregation (KS) model

Variable type	Name	Description	Distribution type	Distribution parameters	Units
Random variable	t^{KS}	Time	Uniform	$lower = 0$, $upper = 100$	sec
Random variable	R^{KS}	Plasma membrane rigidity	Uniform	$lower = 0$, $upper = 100$	$kT \cdot nm^2$
Random variable	Dep^{KS}_{tScale}	Sigmoid height for t parameter	Normal	$\mu = 153.6$, $\sigma = 10.6$	nm
Random variable	Dep^{KS}_{tCen}	Sigmoid center for t parameter	Normal	$\mu = 36.5$, $\sigma = 2.74$	
Random variable	Dep^{KS}_{tDev}	Sigmoid width for t parameter	Normal	$\mu = 23.8$, $\sigma = 2.41$	
Random variable	Dep^{KS}_{RScale}	Sigmoid height for κ parameter	Normal	$\mu = 63.9$, $\sigma = 8.46$	
Random variable	Dep^{KS}_{RCen}	Sigmoid center for κ parameter	Normal	$\mu = 29.2$, $\sigma = 3.24$	
Random variable	Dep^{KS}_{RDev}	Sigmoid width for κ parameter	Normal	$\mu = 9.9$, $\sigma = 2.2$	
Random variable	Dep^{KS}_{output}	Output	Normal	$\mu = \frac{tScale}{1 + e^{-\frac{t - tCen}{tDev}}} + \frac{\kappa Scale}{1 + e^{-\frac{(\kappa - \kappa Cen)}{\kappa Dev}}}$, $\sigma = 20$	

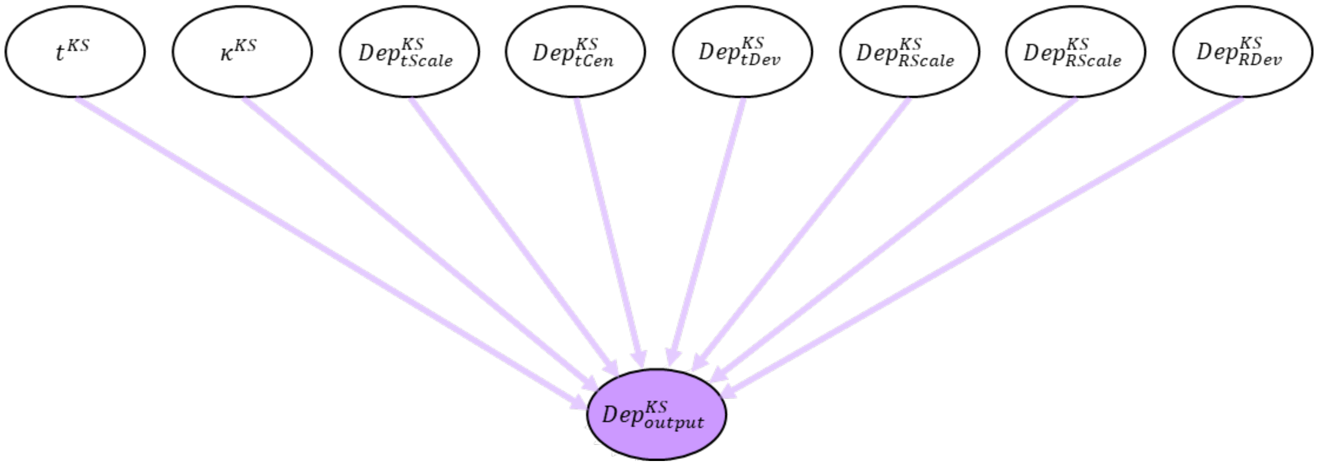

Supplementary Table S3. Random variables for the surrogate KS model after training. top: random variables for the surrogate KS model after training. **Bottom**: Bayesian network for the surrogate KS model after training.

Lck-A model

Model initial configuration: The membrane is represented as a two-dimensional grid of 400 x 400 squares of 10 nm x 10 nm each (**Fig. 2B, left**). At the center of the array there is a CD45 molecule (activating location). Each simulation is also initiated with a single Lck molecule at the center of the array in the active state (Lck*).

Model simulation. We performed 1,000 independent simulations for each parameter combination. In each independent simulation, the Lck* molecule diffuse away from the center with a diffusion coefficient D_{Lck} it is spontaneously deactivated with probability Poff at every iteration (**Fig. 2B, center**). A typical simulation runs for 1000 iterations. To simulate Brownian diffusion of each molecule along the membrane, we sample a molecular step $\boldsymbol{r}$ in a random direction (uniformly sampled between 0 and 2π radians), and with a magnitude that is the absolute value of a normally distributed scalar with mean 0.0 and standard-deviation $\sqrt{4 \cdot D \cdot \Delta t}$ μm; *D* is the diffusion coefficient in units of $\mu m^2 \cdot s^{-1}$. Therefore, we can treat the propagation of the simulation as propagation in time with time step $\Delta t = 0.01\ sec$. We used periodic boundary conditions (molecules that exit at one side enter on the other side). At every iteration all the molecules 'hop' to a new location with no limiting conditions.

Model output: The model output includes the radial distribution function and the decay length for Lck* molecules relative to the CD45 molecule, for the specified set of model parameters (**Fig. 2B, right**). The relative activity of Lck* is normalized to obtain a maximal value of 1.0 when it is near the CD45 molecule.

The surrogate Lck-A model

To learn a surrogate model for the Lck-A model, we first mapped its outputs for different input parameter values (**Fig. 2B**). We then created a probabilistic graphical model that describes statistical relations among its variables in parameterized form (**Fig. 4B,F,I,M; Supp. Tables S4,5**); next, we fitted the probabilistic graphical model to recapitulate the statistical relations between the model parameters and its values (**Supp. Table S4**).

Lck activation (Lck-A) model

Variable type	Name	Description	Distribution type	Distribution parameters	Units
Free parameter	D^{LCK-A}	Diffusion coefficient	Uniform	$\mu = -2$, $\sigma = -1$	$\mu m^2/sec$
Free parameter	$Poff^{LCK-A}$	Probability of deactivation	Uniform	$\mu = -2$, $\sigma = -1$	–
Random variable	DL_{p00}^{LCK-A}	$D^0, Poff^0$	Normal	$\mu = 1.5$, $\sigma = 0.12$	nm
Random variable	DL_{p10}^{LCK-A}	$D^1, Poff^0$	Normal	$\mu = -1$, $\sigma = 0.1$	$nm/(\mu m^2/sec)$
Random variable	DL_{p01}^{LCK-A}	$D^0, Poff^1$	Normal	$\mu = 0.8$, $\sigma = 0.1$	nm
Random variable	DL_{p20}^{LCK-A}	$D^2, Poff^0$	Normal	$\mu = -0.1$, $\sigma = 0.02$	$nm/(\mu m^2/sec)^2$
Random variable	DL_{p11}^{LCK-A}	$D^1, Poff^1$	Normal	$\mu = 0$, $\sigma = 0.02$	$nm/(\mu m^2/sec)$
Random variable	DL_{p02}^{LCK-A}	$D^0, Poff^2$	Normal	$\mu = 0.1$, $\sigma = 0.03$	nm
Random variable	DL_{output}^{LCK-A}	Output	Normal	$\mu = p_{00} + p_{10}D^1 + p_{01}Poff^1 + p_{20}D^2 + p_{11}D^1Poff^1 + p_{02}Poff^2$, $\sigma = 0.5$	nm

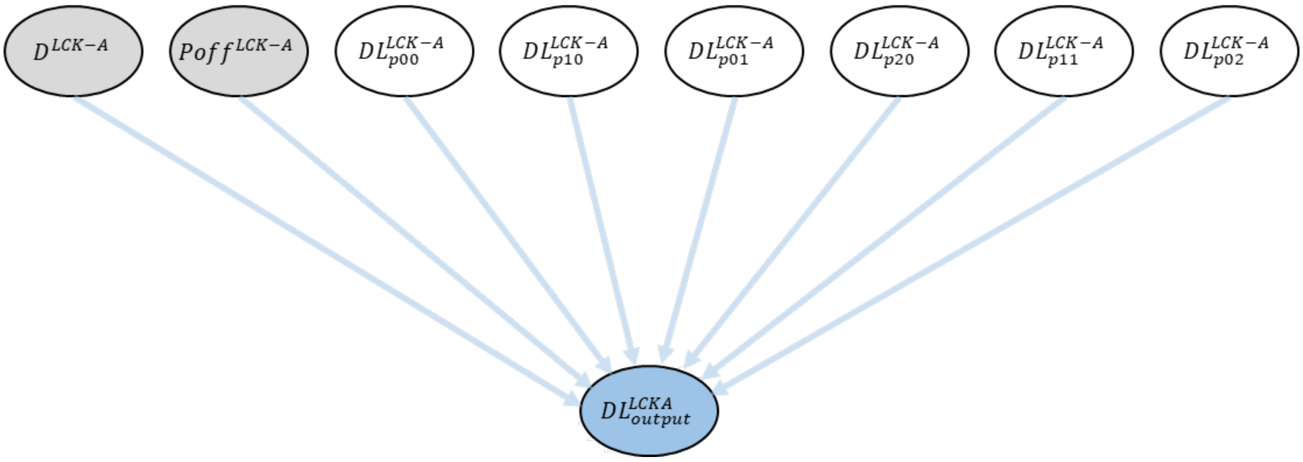

Supplementary Table S4. Random variables for the surrogate Lck-A model before training. top: random variables for the surrogate Lck-A model before training. **Bottom**: Bayesian network for the surrogate Lck-A model before training.

Supplementary Table S5. random variables for the surrogate Lck-A model after training

Lck activation (Lck-A) model

Variable type	Name	Description	Distribution type	Distribution parameters	Units
Random variable	D^{LCK-A}	Diffusion coefficient	Uniform	$\mu = -2$, $\sigma = -1$	$\mu m^2/sec$
Random variable	$Poff^{LCK-A}$	Probability of deactivation	Uniform	$\mu = -2$, $\sigma = -1$	–
Random variable	DL_{p00}^{LCK-A}	$D^0, Poff^0$	Normal	$\mu = 1.5$, $\sigma = 0.12$	nm
Random variable	DL_{p10}^{LCK-A}	$D^1, Poff^0$	Normal	$\mu = -1.01$, $\sigma = 0.069$	$nm/(\mu m^2/sec)$
Random variable	DL_{p01}^{LCK-A}	$D^0, Poff^1$	Normal	$\mu = 0.815$, $\sigma = 0.089$	nm
Random variable	DL_{p20}^{LCK-A}	$D^2, Poff^0$	Normal	$\mu = -0.109$, $\sigma = 0.01$	$nm/(\mu m^2/sec)^2$
Random variable	DL_{p11}^{LCK-A}	$D^1, Poff^1$	Normal	$\mu = 0.06$, $\sigma = 0.014$	$nm/(\mu m^2/sec)$
Random variable	DL_{p02}^{LCK-A}	$D^0, Poff^2$	Normal	$\mu = 0.137$, $\sigma = 0.023$	nm
Random variable	DL_{output}^{LCK-A}	Output	Normal	$\mu = p_{00} + p_{10}D^1 + p_{01}Poff^1 + p_{20}D^2 + p_{11}D^1Poff^1 + p_{02}Poff^2$, $\sigma = 0.5$	nm

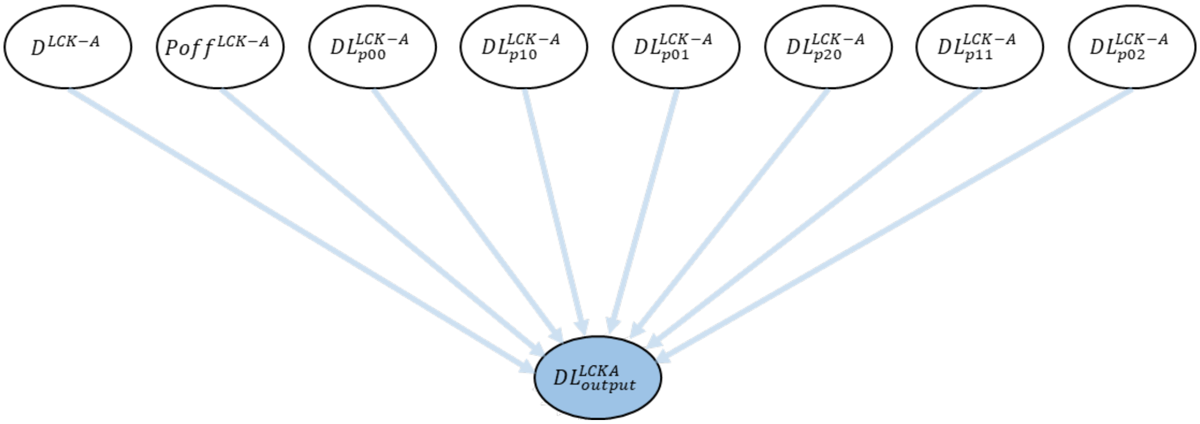

Supplementary Table S5 top: random variables for the surrogate Lck-A model after training. **Bottom**: Bayesian network for the surrogate Lck-A model after training.

pTCR model

Model input: the first input of the model is different point distributions of TCR and CD45 characterized by their depletion distance from the KS model (**Fig. 2C, left**). The second input is the decay-length of the Lck* relative to a CD45 molecule, as described for the Lck-A model above.

Model evaluation: We compute the overall density of Lck* by summing over the Lck* radial distribution functions from the Lck-A model for each individual CD45 molecule (**Fig. 2C, center**). TCRs are then assigned a phosphorylated state if they co-localize with Lck* density beyond a certain threshold (default value of 0.15).

Model output: The first output of the model is the fraction of phosphorylated TCRs (pTCRs) from the total number of TCRs (phosphorylated and unphosphorylated), termed 'Phosphorylation fraction' or 'Phos. frac.'. The second output of the model is the ratio between the 'radius of gyration' of pTCRs to the 'radius of gyration' of all TCRs (phosphorylated and unphosphorylated), termed 'Radius of gyration ratio' or 'Rg ratio' (**Fig. 2C, right**).

Surrogate pTCR model

To learn a surrogate model for the pTCR model, we first mapped its outputs for different input parameter values (**Fig. 2C**). We then created a probabilistic graphical model that describes statistical relations among its variables in parameterized form (**Fig. 4C,H,J,K,N,O; Supp. Tables S6-S9**); next, we fitted the probabilistic graphical model to recapitulate the statistical relations between the model parameters and its values.

TCR phosphorylation (pTCR) model, (Phos. fraction)

Variable type	Name	Description	Distribution type	Distribution parameters	Units
Free parameter	DL^{pTCR}	Decay length	Normal	$\mu = 100$, $\sigma = 50$	nm
Free parameter	Dep^{pTCR}	Depletion	Normal	$\mu = 100$, $\sigma = 50$	nm
Random variable	$Phos_{a}^{pTCR}$	$Intercept$	Normal	$\mu = 0.1$, $\sigma = 0.1$	–
Random variable	$Phos_{DLScale}^{pTCR}$	Sigmoid height for DL parameter	Normal	$\mu = 0.9$, $\sigma = 0.2$	–
Random variable	$Phos_{DLCen}^{pTCR}$	Sigmoid center for DL parameter	Normal	$\mu = 110$, $\sigma = 30$	nm
Random variable	$Phos_{DLDev}^{pTCR}$	Sigmoid width for DL parameter	Normal	$\mu = 40$, $\sigma = 10$	nm
Random variable	$Phos_{DepScale}^{pTCR}$	Sigmoid height for Dep parameter	Normal	$\mu = -0.5$, $\sigma = 0.2$	nm
Random variable	$Phos_{DepCen}^{pTCR}$	Sigmoid center for Dep parameter	Normal	$\mu = 100$, $\sigma = 20$	nm
Random variable	$Phos_{DepDev}^{pTCR}$	Sigmoid width for Dep parameter	Normal	$\mu = 80$, $\sigma = 20$	nm
Random variable	$Phos_{output}^{pTCR}$	Output	Normal	$\mu = a + \frac{DLScale}{1 + e^{-\frac{DL-DLCen}{DLDev}}} + \frac{DepScale}{1 + e^{-\frac{(Dep-DepCen)}{DepDev}}}$, $\sigma = 0.1$	–

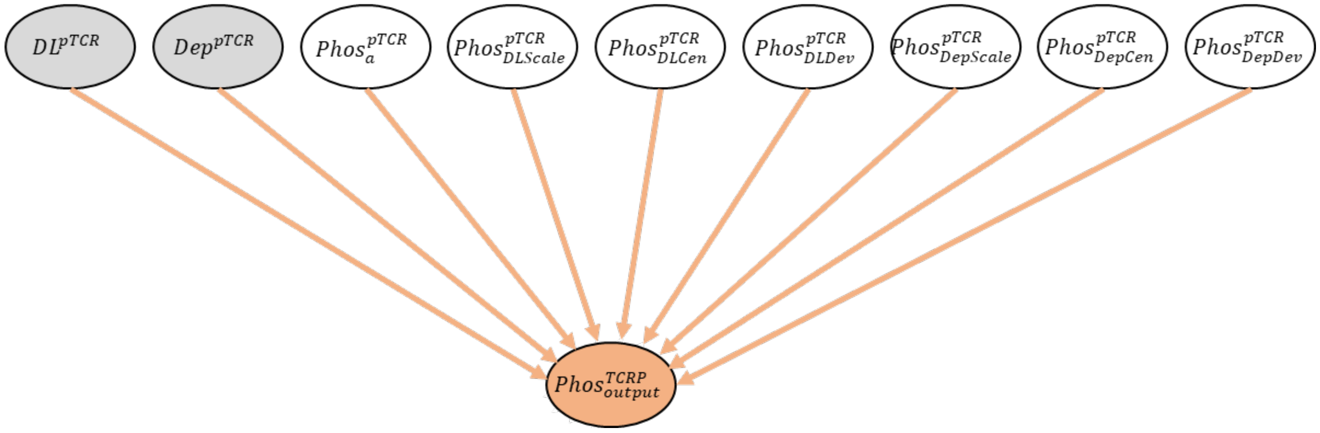

Supplementary Table S6. Random variables for the surrogate Rg ratio pTCR model before training. top: random variables for the surrogate Rg ratio pTCR model before training. **Bottom**: Bayesian network for the surrogate Rg ratio pTCR model before training.

TCR phosphorylation (pTCR) model, (Phos. fraction)

Variable type	Name	Description	Distribution type	Distribution parameters	Units
Random variable	DL^{pTCR}	Decay length	Normal	$\mu = 100$, $\sigma = 50$	nm
Random variable	Dep^{pTCR}	Depletion	Normal	$\mu = 100$, $\sigma = 50$	nm
Random variable	$Phos_{a}^{pTCR}$	*Intercept*	Normal	$\mu = 0.097$, $\sigma = 0.077$	–
Random variable	$Phos_{DLScale}^{pTCR}$	Sigmoid height for DL parameter	Normal	$\mu = 0.924$, $\sigma = 0.116$	–
Random variable	$Phos_{DLCen}^{pTCR}$	Sigmoid center for DL parameter	Normal	$\mu = 111.29$, $\sigma = 15.53$	nm
Random variable	$Phos_{DLDev}^{pTCR}$	Sigmoid width for DL parameter	Normal	$\mu = 41.67$, $\sigma = 7.395$	nm
Random variable	$Phos_{DepScale}^{pTCR}$	Sigmoid height for Dep parameter	Normal	$\mu = -0.507$, $\sigma = 0.121$	nm
Random variable	$Phos_{DepCen}^{pTCR}$	Sigmoid center for Dep parameter	Normal	$\mu = 100.22$, $\sigma = 19.476$	nm
Random variable	$Phos_{DepDev}^{pTCR}$	Sigmoid width for Dep parameter	Normal	$\mu = 82.77$, $\sigma = 18.247$	nm
Random variable	$Phos_{output}^{pTCR}$	Output	Normal	$\mu = a + \frac{DLScale}{1 + e^{-\frac{DL-DLCen}{DLDev}}} + \frac{DepScale}{1 + e^{-\frac{(Dep-DepCen)}{DepDev}}}$, $\sigma = 0.5$	–

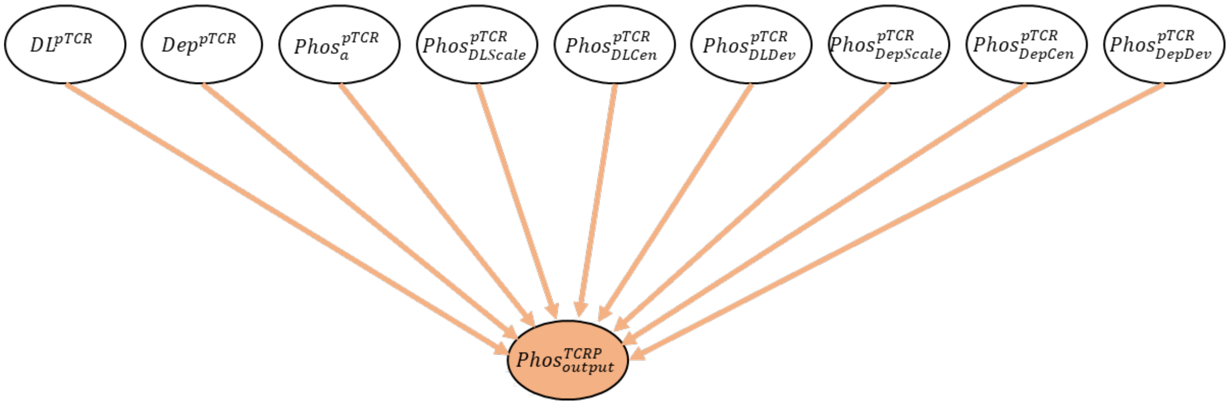

Supplementary Table S7. Random variables for the surrogate Rg ratio pTCR model after training. top: random variables for the surrogate Rg ratio pTCR model after training. **Bottom**: Bayesian network for the surrogate Rg ratio pTCR model after training.

TCR phosphorylation (pTCR) model, (Rg ratio)

Variable type	Name	Description	Distribution type	Distribution parameters	Units
Free parameter	DL^{pTCR}	Decay length	Normal	$\mu = 100$, $\sigma = 50$	nm
Free parameter	Dep^{pTCR}	Depletion	Normal	$\mu = 100$, $\sigma = 50$	nm
Random variable	Rg_a^{pTCR}	$Intercept$	Normal	$\mu = 0.8$, $\sigma = 0.2$	–
Random variable	$Rg_{DLScale}^{pTCR}$	Sigmoid height for DL parameter	Normal	$\mu = 0.9$, $\sigma = 0.2$	–
Random variable	Rg_{DLCen}^{pTCR}	Sigmoid center for DL parameter	Normal	$\mu = 70$, $\sigma = 30$	nm
Random variable	Rg_{DLDev}^{pTCR}	Sigmoid width for DL parameter	Normal	$\mu = -45$, $\sigma = 10$	nm
Random variable	$Rgp_{DepScale}^{pTCR}$	Sigmoid height for Dep parameter	Normal	$\mu = -0.4$, $\sigma = 0.2$	nm
Random variable	Rg_{DepCen}^{pTCR}	Sigmoid center for Dep parameter	Normal	$\mu = 10$, $\sigma = 5$	nm
Random variable	Rg_{DepDev}^{pTCR}	Sigmoid width for Dep parameter	Normal	$\mu = 100$, $\sigma = 20$	nm
Random variable	Rg_{output}^{pTCR}	Output	Normal	$\mu = a + \frac{DLScale}{1 + e^{-\frac{DL-DLCen}{DLDev}}} + \frac{DepScale}{1 + e^{-\frac{(Dep-DepCen)}{DepDev}}}$, $\sigma = 0.1$	–

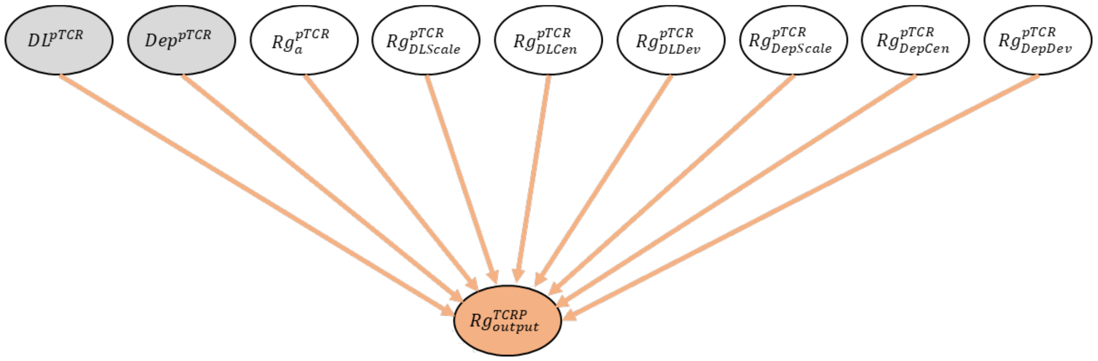

Supplementary Table S8. Random variables for the surrogate Rg ratio pTCR model before training. top: random variables for the surrogate Rg ratio pTCR model before training. **Bottom**: Bayesian network for the surrogate Rg ratio pTCR model before training.

TCR phosphorylation (pTCR) model, (Rg ratio)

Variable type	Name	Description	Distribution type	Distribution parameters	Units
Random variable	DL^{pTCR}	Decay length	Normal	$\mu = 100$, $\sigma = 50$	nm
Random variable	Dep^{pTCR}	Depletion	Normal	$\mu = 100$, $\sigma = 50$	nm
Random variable	Rg_a^{pTCR}	*Intercept*	Normal	$\mu = 0.918$ $\sigma = 0.108$	–
Random variable	$Rg_{DLScale}^{pTCR}$	Sigmoid height for DL parameter	Normal	$\mu = 0.79$ $\sigma = 0.108$	–
Random variable	Rg_{DLCen}^{pTCR}	Sigmoid center for DL parameter	Normal	$\mu = 75.458$ $\sigma = 25.01$	nm
Random variable	Rg_{DLDev}^{pTCR}	Sigmoid width for DL parameter	Normal	$\mu = -51.862$, $\sigma = 8.595$	nm
Random variable	$Rgp_{DepScale}^{pTCR}$	Sigmoid height for Dep parameter	Normal	$\mu = -0.0.17$, $\sigma = 0.131$	nm
Random variable	Rg_{DepCen}^{pTCR}	Sigmoid center for Dep parameter	Normal	$\mu = 9.987$, $\sigma = 54.986$	nm
Random variable	Rg_{DepDev}^{pTCR}	Sigmoid width for Dep parameter	Normal	$\mu = 101.015$, $\sigma = 20.489$	nm
Random variable	Rg_{output}^{pTCR}	Output	Normal	$\mu = a + \frac{DLScale}{1 + e^{-\frac{DL - DLCen}{DLDev}}} + \frac{DepScale}{1 + e^{-\frac{(Dep - DepCen)}{DepDev}}}$, $\sigma = 0.1$	–

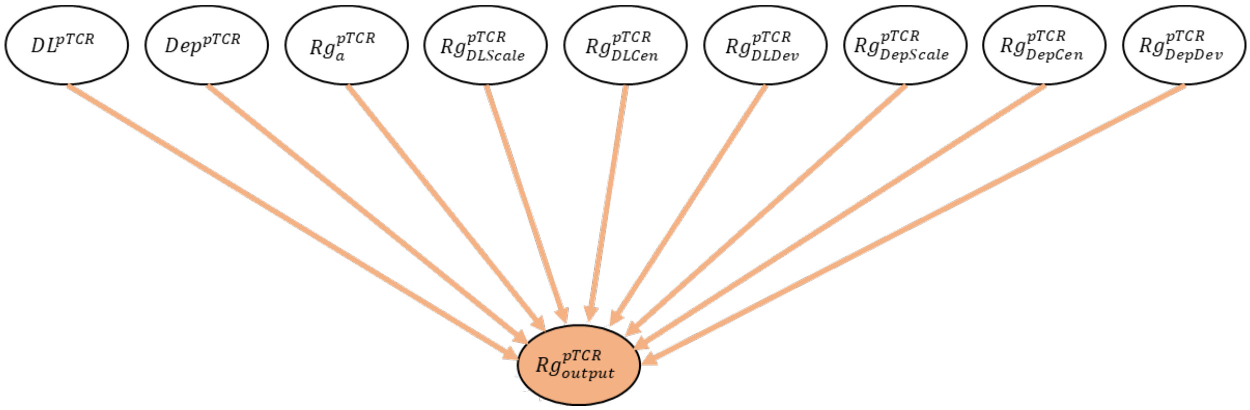

Supplementary Table S9. Random variables for the surrogate Rg ratio pTCR model after training. Top: random variables for the surrogate Rg ratio pTCR model after training. **Bottom**: Bayesian network for the surrogate Rg ratio pTCR model after training.

Csk recruitment model

Model input: The input configurations of the model include point distributions of TCRs, pTCRs, and Lck* on a grid, as in the pTCR model above, without yet considering the effect of Csk recruitment to pTCRs. The TCR activity is initially set to a binary value of either 0.0 (non-phosphorylated TCRs) or 1.0 (pTCRs). For this particular model, we used an Lck-A decay length of 60 nm as the default value, though in principle, other Lck-A decay lengths values could be assessed (see **Fig. 5B,F,I,M**).

Model evaluation: The model operates iteratively until reaching a steady state as follows. First, local Csk relative density is computed proportionally to pTCR phosphorylation level, with an exponential decrease in Csk density about every pTCR molecule; the default value for the Csk exponential decay length is 30 nm, and the maximal density is normalized to a peak of 1.0. Second, the level of Lck* activity is multiplied by the relative density of Csk. Third, Lck* activity is replenished by adding a 0.1 of the difference between its current (low) value and its initial (higher) value, representing the diffusion of “fresh” active Lck* molecules from nearby CD45 molecules. Finally, each pTCR molecule is assigned a probability to be phosphorylated proportionally to local Lck* density (using a proportionality constant with a default value of 0.85). Specifically, repeating this process of de-activation and replenishing for *n*=100 iterations resulted in convergence to the steady state, where Csk recruitment by pTCRs, Lck* de-activation by Csk, and pTCR activation proportionally to Lck* concentrations all balance each other, leading to the output phosphorylation pattern.

Model output: The output of the model is the updated steady-state densities of Lck* and Csk, as well as the phosphorylation probabilities for the various TCR molecules (**Fig. S7C-F**).

Experimental and inferred data used for inferring biophysical parameters from the Bayesian metamodeling

To compute the empirical phosphorylation fraction of TCRs (*Phos.*) and the *Rg ratio (Sections II and III in Results)*, we relied on a dataset of images of isolated T cell contacts (7) as the cells engage activating surfaces (representative examples in **Supp. Fig. S5**). T cell membrane topography was captured using Internal Reflection Microscopy and ZAP-70 molecules were captured using PALM. The IRM values were converted to nm, following calibration and protocol in (7) (see **Fig. 4 and Fig. S2** there). The minimal height in **Supp. Fig. S5** was set to 0 for illustration.

Localizations of TCR and CD45 molecules (in **Figs. 1D, 7A**) were estimated based on the measured topography of the cell. Specifically, we used a height range of 10-15 nm above the surface in which TCR molecules were randomly distributed with an estimated density of 1000 molecules/μm^2, based on empirical measurements (5). The density followed a half-Gaussian distribution with a peak at 0 and a sigma of 5nm. Effectively, such a distribution diminishes at a height of ~13nm. TCR molecules were also placed at locations of Zap-70 within the specified height range (following height contours of 10nm and below; depicted as white contours in **Supp. Fig. S5**) and marked as phosphorylated. CD45 molecules were distributed using a height range of 45-55 nm with a similar density.

Mapping the effect of pMHC:TCR association strength on pTCR dynamics

To assess the effects of pMHC:TCR association strengths and Lck decay length values on the extent and dynamics of TCR phosphorylation, we applied all three partial models in our metamodel, similarly to our previous analyses (**Figures 2-8**). To modulate the pMHC:TCR association strength parameter, we modify the corresponding binding parameter U_{assoc} in the KS model (**Supp. Table S1; Figure 2**, top right, binding potential in green). For simplicity, we refer to this parameter as U from hereon. Unlike previous analyses in this study, here, the coupling between the partial model was not done probabilistically via the surrogate models, but rather through direct propagation of the outputs for the KS model and Lck-A model as inputs to the pTCR model (**Figure 6**).

Initial contact formation. Our simulations focused on a specific and simplified scenario of an initial contact has formed between the T cell and an APC (8). To generate the initial configurations for a pre-formed contact, we first simulated the system in a constrained setting for 10 seconds using the KS model. (t = 0), TCR molecules were positioned in a circular cluster with a diameter of 400 nm and a density of 1000/μm^2 (n ~ 120). CD45 molecules were positioned in a circular cluster with a diameter of 800 nm and a density of 1000/μm^2 (n ~ 480). We initially constrained the inter-membrane distance to 70 nm, except at the locations of molecules, where the membrane distance was constrained to either 13 nm for pMHC:TCR (promoting their binding), or 50 nm for CD45 (accommodating their larger size). These initial distances were kept fixed during the 10 seconds (i.e. first 1000 iterations of the KS model), resulting in a stochastic initial configuration for the pre-formed contact. During this period, the molecules could diffuse and interact, and the membrane gradually flattened.

Simulations of TCR unbinding and phosphorylation dynamics. Starting from **the** pre-formed contact, the imposed constraint on the inter-membrane distance was lifted for the KS model, effectively allowing the pMHC:TCR complexes to more easily detach, letting the TCR and CD45 molecules diffuse more freely and the inter-membrane distance to change as the contact evolved. The simulations ran for an additional 90 seconds (9,000 iterations), during which we monitored the extent of TCR binding and phosphorylation by using the outputs of the KS model and the Lck-A models as inputs to the pTCR model in our metamodel (**Fig. 2, 5**). We note that in the pTCR model, unbound TCRs cannot be phosphorylated even if they coincide with a high density of Lck. In other words, the number of bound TCR molecules imposes an upper limit on the number of phosphorylated TCR molecules.

Independent simulations over a range of pMHC:TCR association strengths and Lck decay lengths. We performed a total of 900 independent simulations. In each simulation, the association strength between the TCRs and the pMHC molecules, U, was kept constant and was scanned between 5 and 50 KT in different simulations (5 independent simulations per each U) at 5 kT intervals (U = 5, 10, …, 45, 50 kT). The transition region between U=5 kT and U=35 kT was mapped at a 1 kT interval (U=25, 26, …, 34, 35 kT). The decay length of active Lck was considered for the following values: 10 to 100 nm at 10 nm intervals.

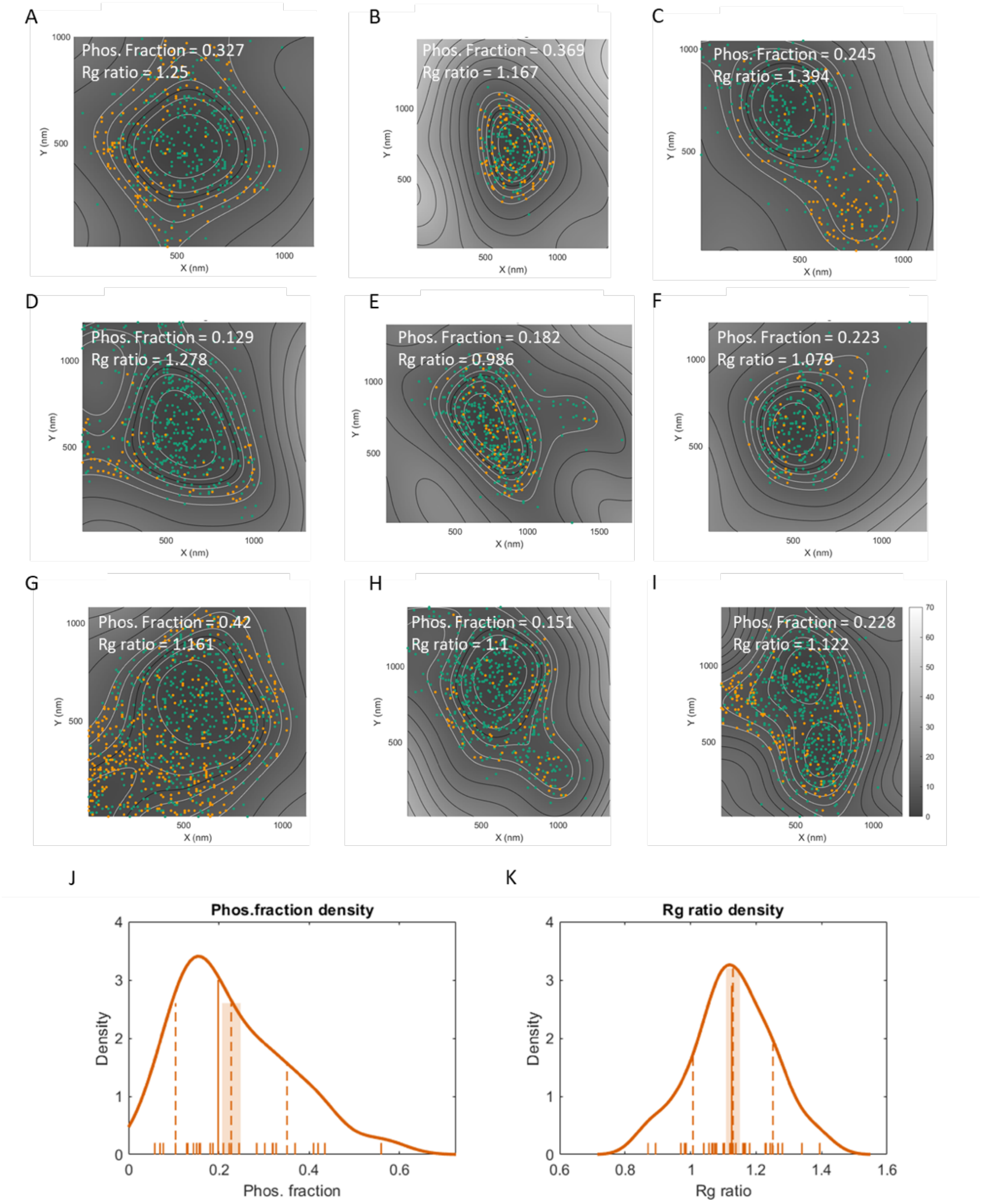

Supplementary Figure S5. Representative experimental images of T cell membrane patches showing Zap-70 patterning at early T cell contacts. **A-I.** The shown contacts are zoom images of representative isolated T cell contacts [data from (7)]. T cell membrane topography was captured using Internal Reflection Microscopy (gray contour maps) and ZAP-70 molecules (orange points) were captured using PALM (7). The TCR molecules (green) are inferred from the membrane topography based on prior information on TCR distributions at APC contact regions (**Supp. Methods**). The difference between consecutive contours corresponds to a difference of 2 nm in the membrane height for the white contours and 5 nm for the black contours (7), with dark colors representing “valleys” in the membrane topography, and black being a local minimum corresponding to the tight contact areas between the T cell and the activating surface. The ZAP-70 molecules are enriched at the periphery of these tight contact areas. **J.** Distribution of Phosphorylation fraction values (N=29 contacts). **K.** Distribution of the Rg ratio values (N=29 contacts). Dashed vertical lines in B and C mark standard-deviations of the distribution around the mean. The median is depicted in solid vertical line, and the SEM is shaded around the mean.

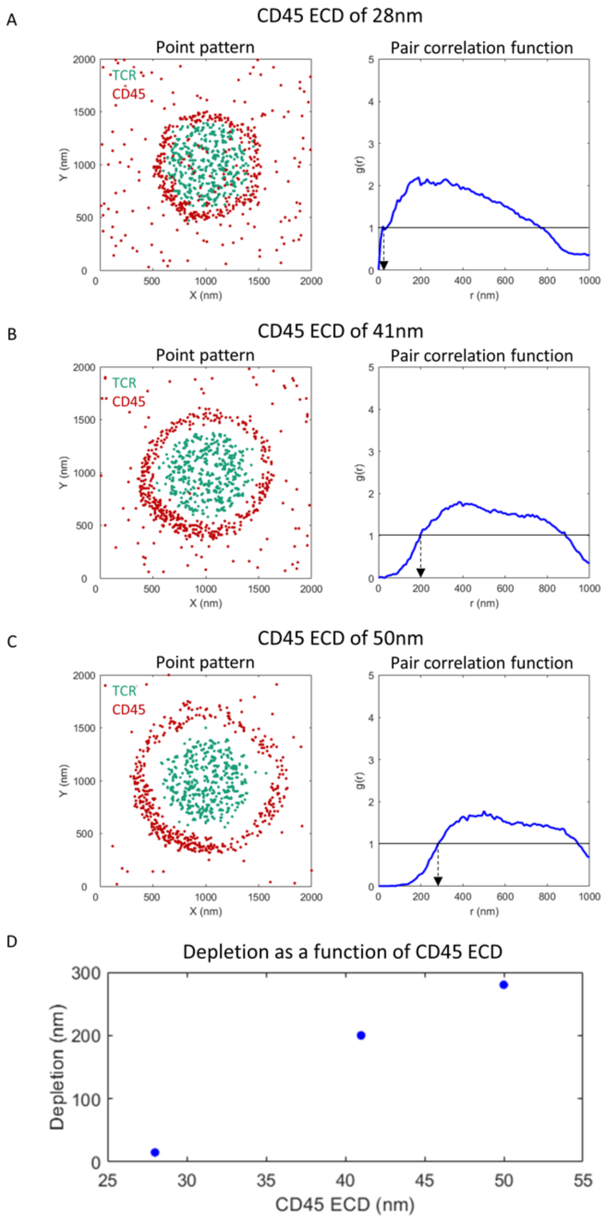

Supplemental Figure S6. Longer CD45 isoforms enlarge the depletion length from TCR. Simulation results using InterCells, showing the molecular patterning of TCR and three different CD45 isoforms within the contact of a T cell and an APC. The ectodomain (ECD) size of the isoforms was 28, 41 and 50 nm (9,10). **A-C.** Right, The patterning after 100 sec. (as in Fig. 2A). Left, the bivariate pair-correlation function of TCR and the CD45 isoforms. **D.** Depletion length *vs.* the size of the isoform ECD.

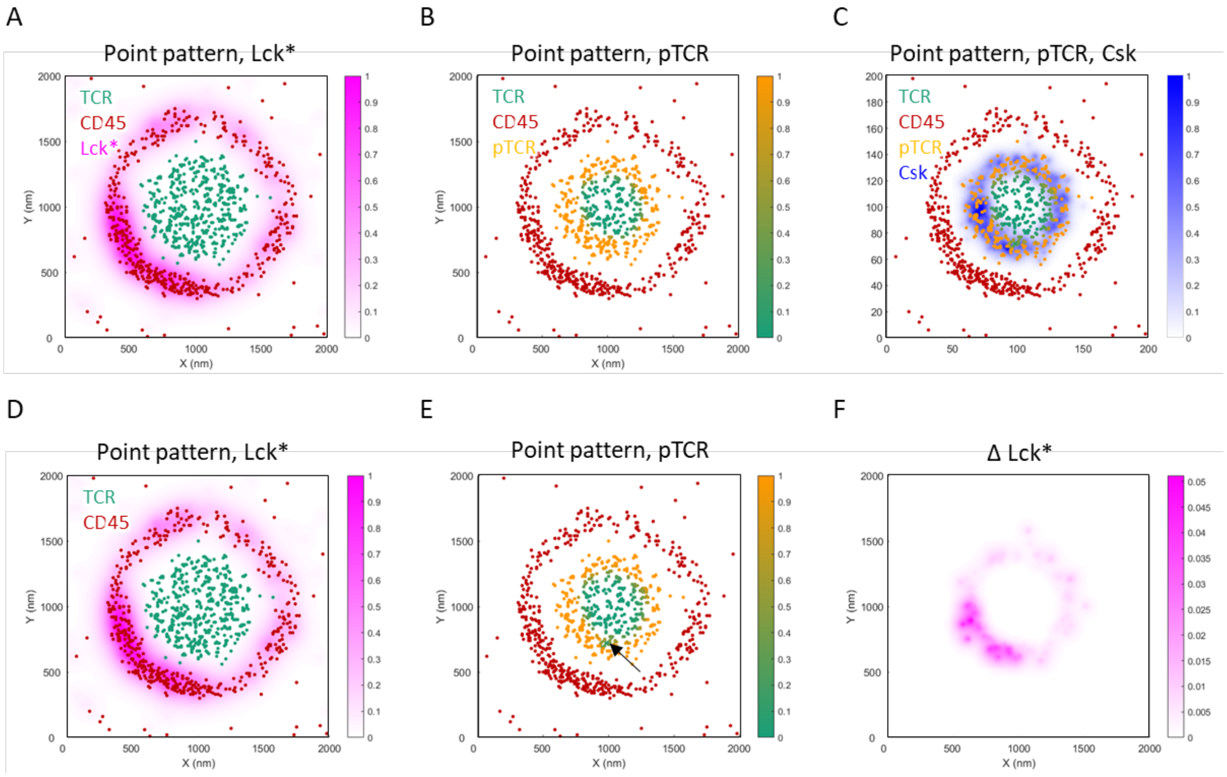

Supplemental Figure S7. Csk recruitment model and its effect on pTCR localization and Lck* density. **A.** Point pattern of TCR and CD45 molecules computed using the KS model. The density of Lck* molecules computed using the Lck-A model for the various CD45 molecules is shown in magenta**. B.** The same point pattern as in A, colored by fractional TCR activation (green, non-phosphorylated to yellow, fully phosphorylated), computed from Lck* similarly to the pTCR model, without considering Csk recruitment. **C-F.** Results for the same point pattern as in A when considering the Csk recruitment model. **C.** pTCR/TCR/CD45 localization and activity level as in B and Csk activity level from low (white) to high (blue) at the steady state following Csk recruitment. **D.** The same point pattern distribution as in A, with the steady-state density of Lck* following Csk recruitment and its deactivation of Lck*. **E.** The steady-state TCR phosphorylation pattern as in B following Csk recruitment. A local reduction in the TCR phosphorylation level due to Csk recruitment is indicated using a black arrow (compare to **B**). **F.** The local difference in Lck* density between panel D and panel A. Csk recruitment enhances the edge effect of pTCRs at the periphery of the TCR cluster. Color-bars represent relative densities of Lck* (in panels **A,D**), DLck*(**F**), pTCR (**B,E**) and Csk (**C**).

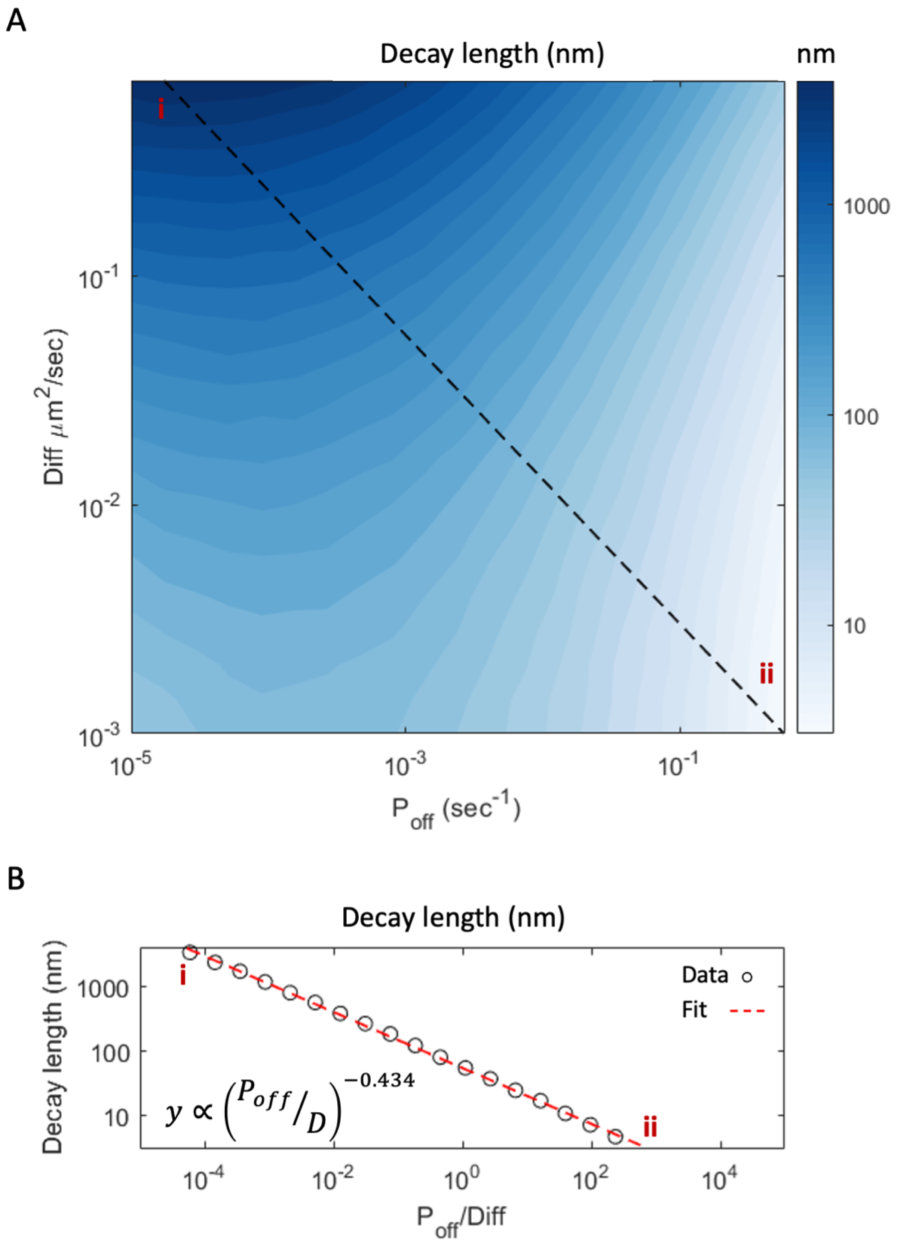

Supplemental Figure S8. Dependency of decay length (activity range) of active Lck on the ratio between the diffusion coefficient of Lck *Diff* and the de-activation rate P_{off}. A Cross section of parameter values for which the decay length in B was computed as a function of P_{off} /Diff, which increases from top-left (i) to bottom-right (ii), as in Fig. 4. **B**. Decay length as a function of P_{off} /Diff along the cross-section in A, with a linear fit in log-log scale corresponding to the power law relating the decay length (y axis) with the approximate inverse square root of P_{off} /Diff (x axis). The units of P_{off} /Diff are specified in μm^{-2} . (i) and (ii) are as in **A**.

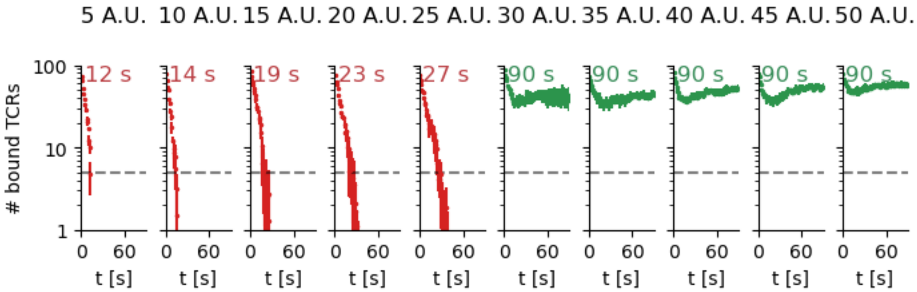

Supplemental Figure S9. Number of bound TCRs as a function of time for different pMHC:TCR association strengths (columns).

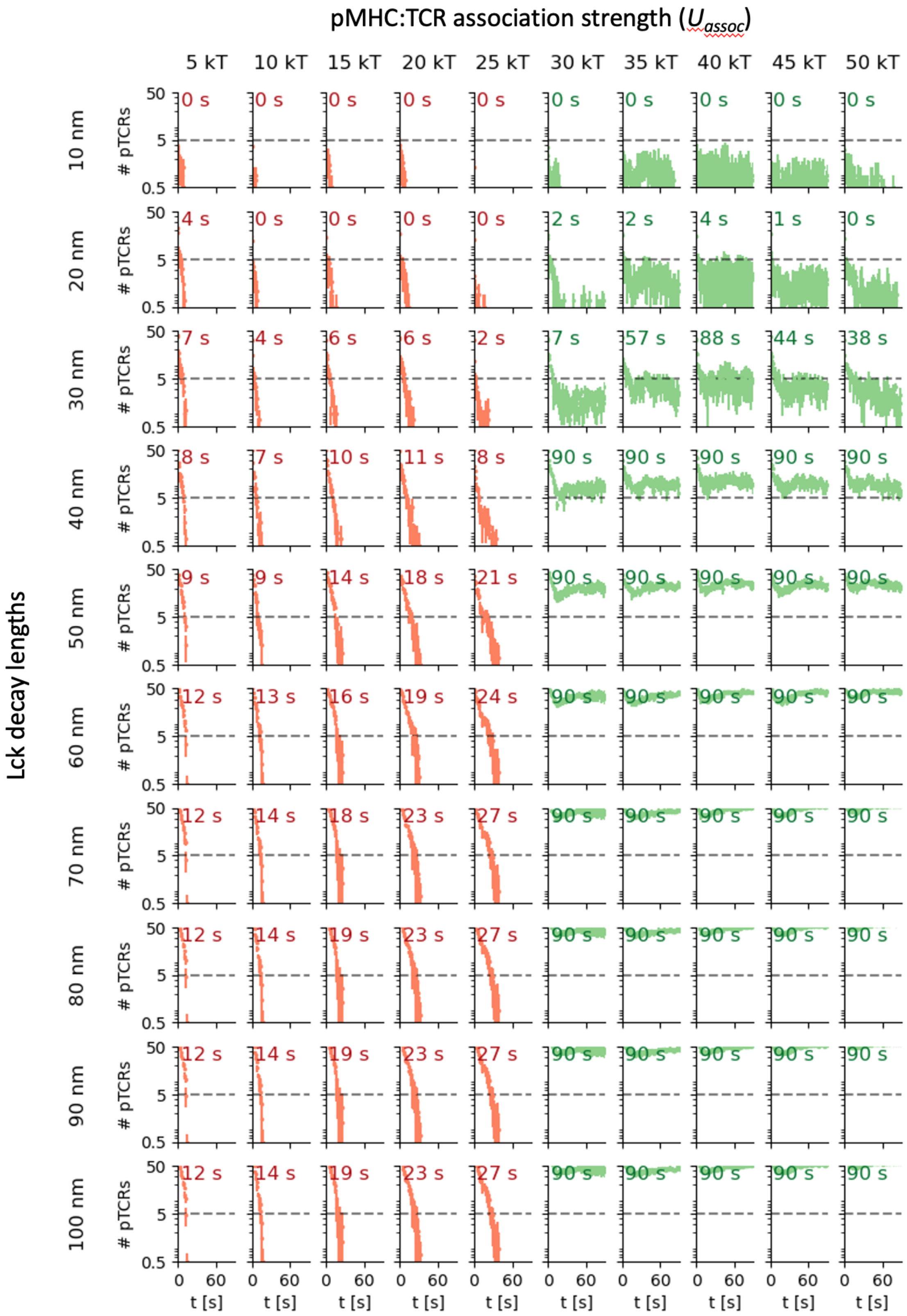

Supplemental Figure S10. Phosphorylated TCRs as a function of time for different pMHC:TCR association strengths (columns) and Lck decay lengths (rows).

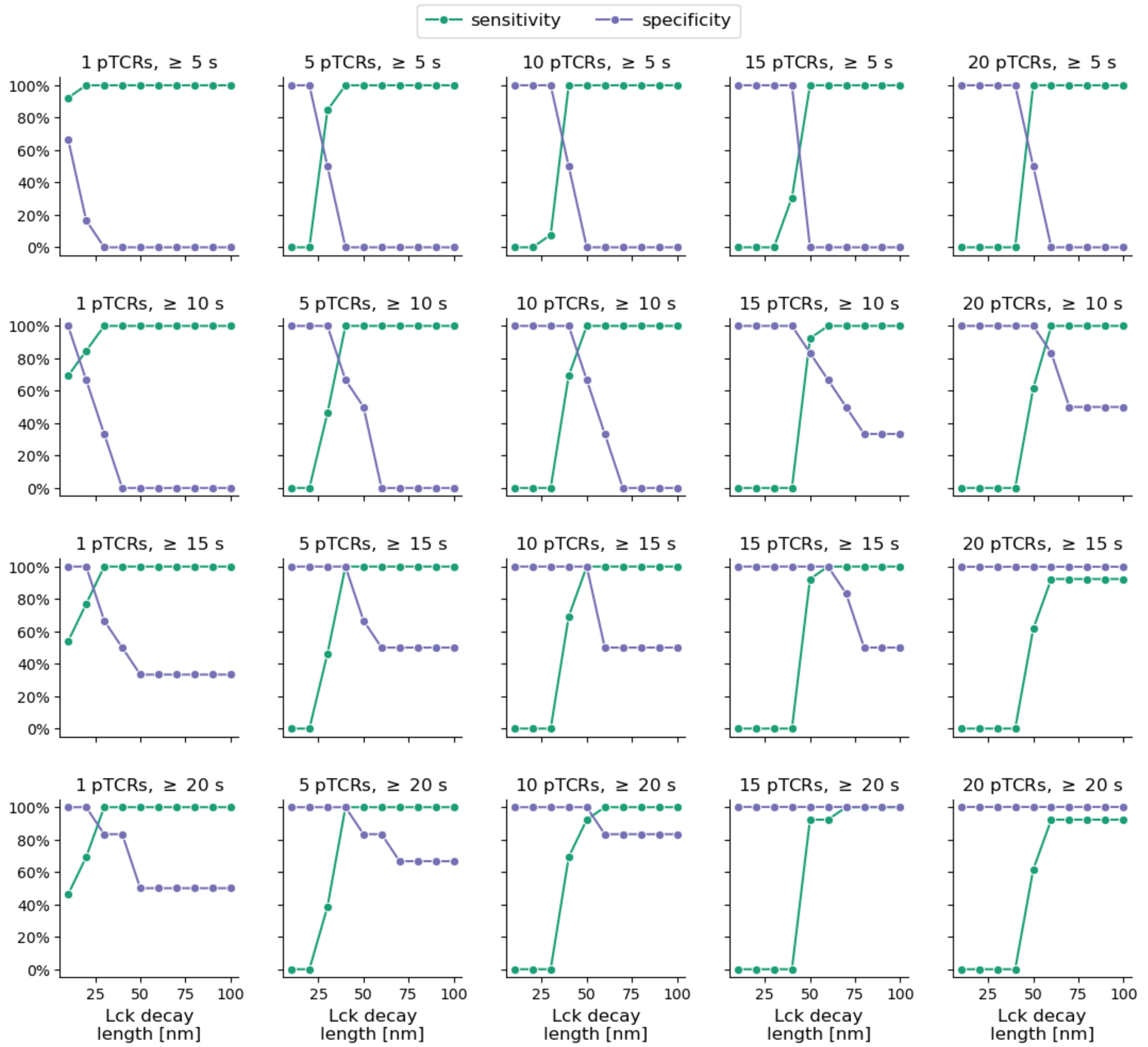

Supplemental Figure S11. Sensitivity (green) and specificity (purple-blue) in a model where early T cell activating requires at least a certain number of TCRs (columns) to be phosphorylated for a duration of at least a certain number of seconds (rows). Each point in the curves shown is computed for all pMHC:TCR association strengths under study ($U = 5 - 50\ kT$), with a threshold of 25 kT for distinguishing low and high association strengths as hypothetical negative and positive ground-truth results, respectively.

Supplementary References

1. Salvatier J., Wiecki T.V., Fonnesbeck C. Probabilistic programming in Python using PyMC3. *PeerJ Computer Science* (2016) 2:e55. doi: 10.7717/peerj-cs.55

2. Hoffman MD, Gelman A. The no-U-Turn Sampler: Adaptively setting path lengths in Hamiltonian Monte Carlo. *arXiv [statCO]* (2011) https://www.jmlr.org/papers/volume15/hoffman14a/hoffman14a.pdf

3. Weikl TR, Asfaw M, Krobath H, Różycki B, Lipowsky R. Adhesion of membranesviareceptor–ligand complexes: Domain formation, binding cooperativity, and active processes. *Soft Matter* (2009) 5:3213–3224. doi: 10.1039/B902017A

4. Hastings WK. Monte Carlo sampling methods using Markov chains and their applications. *Biometrika* (1970) 57:97–109. doi: 10.1093/biomet/57.1.97

5. Neve-Oz Y, Sajman J, Razvag Y, Sherman E. InterCells: A Generic Monte-Carlo Simulation of Intercellular Interfaces Captures Nanoscale Patterning at the Immune Synapse. *Front Immunol* (2018) 9:2051. doi: 10.3389/fimmu.2018.02051

6. Raveh B, Sun L, White KL, Sanyal T, Tempkin J, Zheng D, Bharath K, Singla J, Wang C, Zhao J, et al. Bayesian metamodeling of complex biological systems across varying representations. *Proc Natl Acad Sci U S A* (2021) 118: doi: 10.1073/pnas.2104559118

7. Razvag Y, Neve-Oz Y, Sajman J, Yakovian O, Reches M, Sherman E. T Cell Activation through Isolated Tight Contacts. *Cell Rep* (2019) 29:3506-3521.e6. doi: 10.1016/j.celrep.2019.11.022

8. Cai E, Marchuk K, Beemiller P, Beppler C, Rubashkin MG, Weaver VM, Gérard A, Liu T-L, Chen B-C, Betzig E, et al. Visualizing dynamic microvillar search and stabilization during ligand detection by T cells. *Science* (2017) 356: doi: 10.1126/science.aal3118

9. McCall MN, Shotton DM, Barclay AN. Expression of soluble isoforms of rat CD45. Analysis by electron microscopy and use in epitope mapping of anti-CD45R monoclonal antibodies. *Immunology* (1992) 76:310–317.

10. Chang VT, Fernandes RA, Ganzinger KA, Lee SF, Siebold C, McColl J, Jönsson P, Palayret M, Harlos K, Coles CH, et al. Initiation of T cell signaling by CD45 segregation at “close contacts.” *Nat Immunol* (2016) 17:574–582. doi: 10.1038/ni.3392
